# Antibody-mediated depletion of CCR10^+^EphA3^+^ cells ameliorates fibrosis in IPF

**DOI:** 10.1172/jci.insight.141061

**Published:** 2021-06-08

**Authors:** Miriam S. Hohmann, David M. Habiel, Milena S. Espindola, Guanling Huang, Isabelle Jones, Rohan Narayanan, Ana Lucia Coelho, Justin M. Oldham, Imre Noth, Shwu-Fan Ma, Adrianne Kurkciyan, Jonathan L. McQualter, Gianni Carraro, Barry Stripp, Peter Chen, Dianhua Jiang, Paul W. Noble, William Parks, John Woronicz, Geoffrey Yarranton, Lynne A. Murray, Cory M. Hogaboam

**Affiliations:** 1 Women’s Guild Lung Institute, Division of Pulmonary and Critical Care Medicine, Department of Medicine, Cedars-Sinai Medical Center, Los Angeles, California, USA.; 2 Division of Pulmonary, Critical Care and Sleep Medicine, Department of Internal Medicine, University of California, Davis, Sacramento, California, USA.; 3 Division of Pulmonary and Critical Care Medicine, Department of Medicine, School of Medicine, University of Virginia, Charlottesville, Virginia, USA.; 4 KaloBios Pharmaceuticals, Inc. (now Humanigen, Inc.), Burlingame, California, USA.; 5 MiroBio Ltd, Oxford, United Kingdom.

**Keywords:** Pulmonology, Fibrosis

## Abstract

Idiopathic pulmonary fibrosis (IPF) is characterized by aberrant repair that diminishes lung function via mechanisms that remain poorly understood. CC chemokine receptor (CCR10) and its ligand CCL28 were both elevated in IPF compared with normal donors. CCR10 was highly expressed by various cells from IPF lungs, most notably stage-specific embryonic antigen-4–positive mesenchymal progenitor cells (MPCs). In vitro, CCL28 promoted the proliferation of CCR10^+^ MPCs while CRISPR/Cas9–mediated targeting of CCR10 resulted in the death of MPCs. Following the intravenous injection of various cells from IPF lungs into immunodeficient (NOD/SCID-γ, NSG) mice, human CCR10^+^ cells initiated and maintained fibrosis in NSG mice. Eph receptor A3 (EphA3) was among the highest expressed receptor tyrosine kinases detected on IPF CCR10^+^ cells. Ifabotuzumab-targeted killing of EphA3^+^ cells significantly reduced the numbers of CCR10^+^ cells and ameliorated pulmonary fibrosis in humanized NSG mice. Thus, human CCR10^+^ cells promote pulmonary fibrosis, and EphA3 mAb–directed elimination of these cells inhibits lung fibrosis.

## Introduction

Idiopathic pulmonary fibrosis (IPF) is an interstitial lung disease characterized by chronic and progressive scarring of the lungs associated with inexorable decline in lung function. It is one of the most aggressive forms of interstitial pneumonias, with a median time of survival of 3–5 years after diagnosis (1). IPF is defined on the basis of the presence of a radiological and/or histological pattern of usual interstitial pneumonia in the absence of an alternate etiology for this pattern. Features include excessive deposition of collagen and extracellular matrix, which results in heterogeneous fibrosis and loss of normal lung architecture, with or without honeycomb cyst formation (2). The links between genetics and environment in IPF have been extensively investigated (3–7). However, none of these studies have consistently identified a causative agent(s) in IPF, thereby making it difficult to accurately model and validate novel therapeutics in this disease. Adding to this complexity is the fact that lymphocytes (8, 9); myeloid (10–13), epithelial (14, 15), endothelial (16–18), and mesenchymal cells (19–23); as well as several signaling pathways propagated in these cells (9, 24, 25) have all been implicated in disease progression and/or prognosis in IPF. Although 2 therapeutics have been approved for the treatment of IPF patients, both Esbriet/pirfenidone (26–28) and Ofev/nintedanib (29–31) only slow but do not halt the progression of this disease. Thus, research efforts have been directed at cell-mediated pathways, which are not targeted by these drugs and might contribute to IPF progression.

Although the mechanisms driving progression in IPF are still not fully understood, there is growing evidence that chemokines and their receptors modulate the progression of lung fibrosis (32–38). Specifically, we recently identified a novel CCR10^+^ epithelial cell population in IPF lungs. These cells promoted lung remodeling in humanized NSG mice, and their numbers positively correlated with lung hydroxyproline levels (39). Despite this novel role of CCR10^+^ epithelial cells in driving lung remodeling, further characterization studies revealed that their contribution to matrix deposition was most likely indirect via activation of lung fibroblasts. Thus, in the present study, we have investigated the role of CCR10 pathway in fibroblasts and other cell types that may directly contribute to pulmonary fibrosis. Moreover, we have explored a potentially novel targeting approach of CCR10^+^ cells as a means of preventing and/or ameliorating lung fibrosis.

## Results

### CCR10 expression is significantly increased in rapidly progressive IPF.

**Figure 1:**
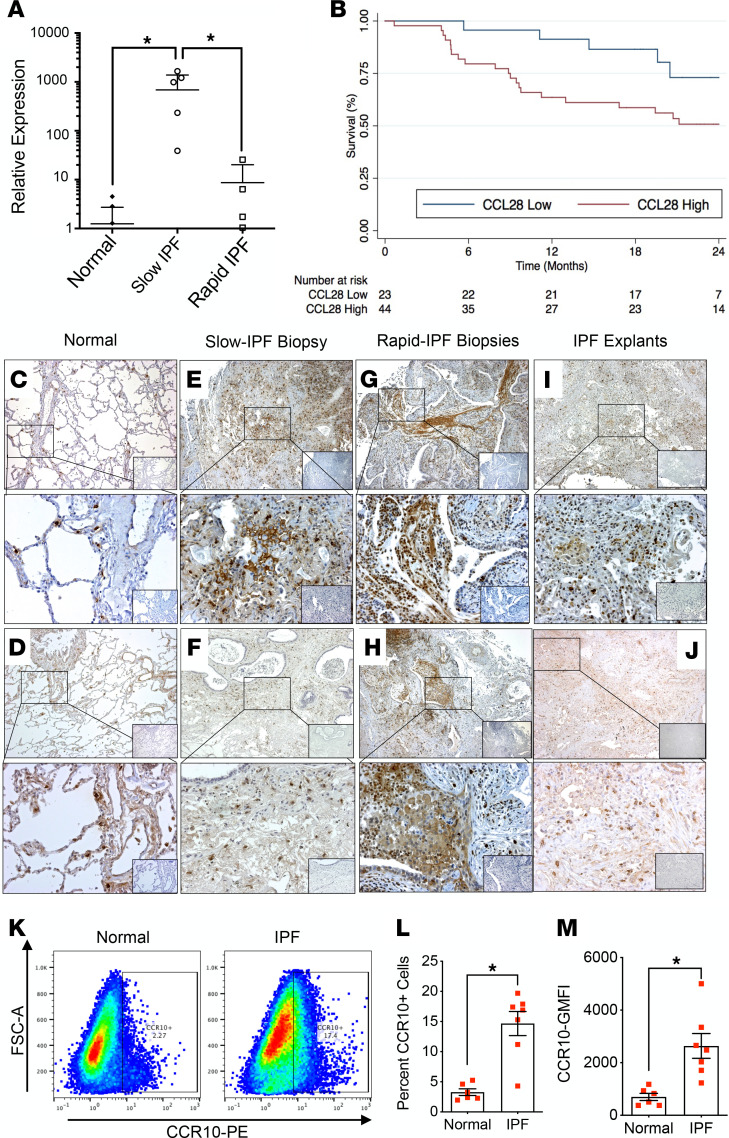
CCR10 expression in normal and IPF lungs. (**A**) Transcriptomic analysis of lung samples (normal and rapid- and slow-IPF) for CCR10 transcripts. Data shown are mean ± SEM; *n* = 3–9/group. *P ≤ 0.05 via 1-way ANOVA with Tukey’s multiple comparisons test. (**B**) Analysis of peripheral blood CCL28 transcript expression and progression-free survival, as defined by death or 10% forced vital capacity (FVC) decline in 67 IPF patients over 24 months. (**C**–**L**) IHC analysis for CCR10 protein expression in normal lung explants (**C** and **D**), slow-IPF (**E** and **F**) and rapid-IPF lung biopsies (**G** and **H**), and IPF lung explants (**I** and **J**). Shown are representative images acquired at original magnification 50× (top) and 200× (bottom). The corresponding IgG isotype control staining is shown in the inlaid images. *n* = 3–15 lung samples/group. (**K**–**M**) Flow cytometric analysis of cultured fibroblasts from normal and IPF lung explants for cell surface CCR10 protein. Representative dot plots of CCR10 expression (**K**), average percentage of fibroblasts expressing CCR10 (**L**), and the geometric mean fluorescence intensity (GMFI) of CCR10 expression (**M**) on fibroblasts from normal (*n* = 6) and IPF (*n* = 7) lung explants. Data shown are mean ± SEM; *P ≤ 0.05 via 2-tailed Mann-Whitney nonparametric test.

CCR10 transcripts were significantly elevated in IPF biopsies from rapidly progressive patients (i.e., rapid-IPF) compared with either slow-IPF lung biopsies or normal donor lung samples (Figure 1A). Further, survival analysis showed that peripheral blood CCL28 (a CCR10 ligand) transcript levels were correlated (*P* < 0.05) with poor progression-free survival (Figure 1B) and Cox regression unadjusted or adjusted for sex, age, and physiological (SAP) score (Table 1) in the COMET IPF cohort (40, 41). Immunohistochemical (IHC) analysis showed that CCR10^+^ interstitial cells were rarely observed in normal lung explants (Figure 1, C and D) but were most abundant in rapid-IPF lung biopsies (Figure 1, G and H) compared with slow-IPF lung biopsies and IPF lung explants (mainly from slow-IPF patients) (Figure 1, E, F I, and J, respectively).

Flow cytometric analysis of CD45^+^ and CD45^–^ cells from IPF lung explants and control lungs revealed that within the CD45^+^ immune cells few CD68^+^ cells expressed CCR10 (Supplemental Figure 1, A and B; supplemental material available online with this article; https://doi.org/10.1172/jci.insight.141061DS1), but there was a significant increase in the percentage of CCR10-expressing CD3^+^CD4^+^ helper T cells (Supplemental Figure 1, C and D), CD3^+^CD8^+^ cytotoxic T cells (Supplemental Figure 1, C and E), and CD19^+^ B cells (Supplemental Figure 1, F and G) from IPF lungs compared with normal lungs. Analysis of CD45^–^ revealed a significant increase in CCR10^+^ and geometric mean fluorescence intensity (GMFI) (Supplemental Figure 1, H–J) of CCR10 on IPF cells compared with normal lung cells. Considering the significantly higher percentage of CD45^–^CCR10^+^ and the critical role of fibroblasts in lung fibrosis (37, 42, 43), we further assessed CCR10 expression by cultured fibroblasts from lung explants and confirmed increased percentage of CCR10^+^ fibroblasts (Figure 1, K and L) and GMFI (Figure 1M) of CCR10 in IPF cultures compared with normal cultures.

### CCL28 is expressed by alveolar type II epithelial cells, CD68^+^ cells, and fibroblasts in normal and IPF lungs.

The increased percentage of CCR10^+^ cells was associated with the increased presence of its ligand CCL28 in IPF compared with normal lungs. Although CCR10 can bind to CCL27 and CCL28 ligands, CCL27 was not present in gene array data sets of normal and IPF lungs. CCL28, on the other hand, was present in normal alveolar type II epithelial (AT2) cells and to a lesser extent in IPF club and goblet epithelial cells (Supplemental Figure 2, A–C). CCL28 protein was also detected in cultured primary airway epithelial cells derived from the lungs of normal and IPF patients (39) but was higher in the latter group (Supplemental Figure 2D). CCL28 was also detected in the supernatants of normal and IPF fibroblasts (Supplemental Figure 2E). Interestingly, senescent IPF fibroblasts produced significantly more CCL28 compared with senescent normal cells. IHC analysis on serial sections from normal (Supplemental Figure 3, A, B, E, F, I, J M, and N) and IPF (Supplemental Figure 3, C, D, G, H, K, L, O, and P) lung explants showed that CCL28^+^ cells were diffusely localized in normal lungs (Supplemental Figure 3, A and B) and localized in focal regions in IPF lung tissues (Supplemental Figure 3, C and D). Further, these cells localized to areas with CD68 (Supplemental Figure 3, E–H) and/or surfactant protein C–expressing (Supplemental Figure 3, I–L) cells in both normal and IPF lungs. Thus, AT2 cells, CD68^+^ immune cells, and pulmonary fibroblasts appeared to be sources of CCL28 in normal and IPF lungs.

### IPF lung fibroblasts express CCR10 and CCL28 induces minor activation.

The expression and role of this receptor were next assessed in cultured normal and IPF lung fibroblasts. CCR10 was most abundantly expressed among the chemokine receptors analyzed, including CCR7, CXCR1, and CXCR4, in cultured IPF lung fibroblasts (Supplemental Figure 4A). CCL28 promoted a 2-fold increase in collagen type I levels in normal and IPF fibroblasts (Supplemental Figure 4B), although it did not reach statistical significance in the normal lines. CCL28 did not alter α–smooth muscle actin (αSMA) protein levels, IL-6 secretion, or the expression of fibrosis-related genes *COL1A1*, *COL3A1*, and *FN1* in either normal or IPF primary lung fibroblasts (Supplemental Figure 4, C–F). Together, these data suggested that CCL28/CCR10 interactions had a minor effect on the activation of IPF fibroblasts.

### CCR10^+^ IPF cells express the tyrosine kinase receptor Eph receptor A3.

**Figure 2:**
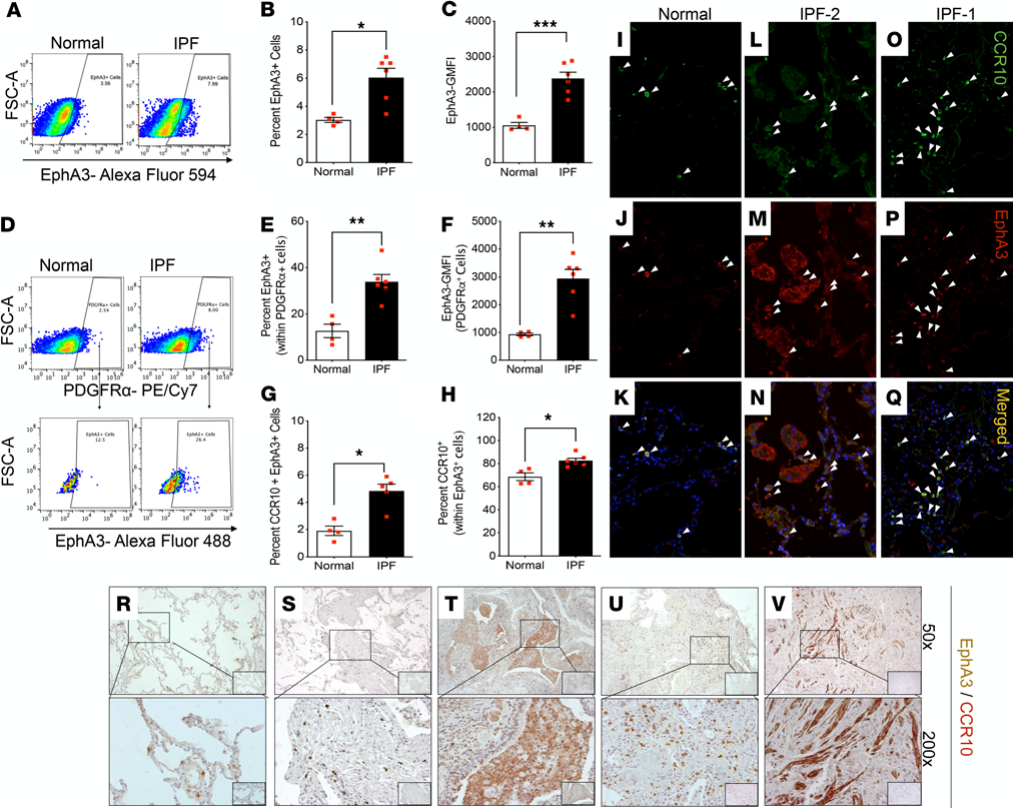
EphA3 expression by fibroblasts and CCR10^+^ cells in normal and IPF lung explants. (**A**–**H**) Flow cytometric analysis of cultured lung fibroblasts from normal and IPF patients for cell surface expression of EphA3, CCR10, stage-specific embryonic antigen-4 (SSEA4), and PDGFRα. Representative dot plot (**A**) and respective percentage of EphA3^+^ cells (**B**) and GMFI of EphA3 (**C**). Representative dot plots (**D**) and respective quantitation of percentage of EphA3^+^ (Alexa Fluor 594) cells (**E**) and GMFI of EphA3 (**F**) within gated PDGFRα^+^ (PE/Cy7) cells. Percentage of CCR10^+^EphA3^+^ cells (**G**) and CCR10^+^ within gated EphA3^+^ cells (**H**). Data shown are mean ± SEM; *n* = 4–6/group; *P ≤ 0.05, **P ≤ 0.01, or ***P ≤ 0.005 via 2-tailed Mann-Whitney nonparametric test. (**I**–**Q**) Representative immunofluorescence images showing CCR10 (green; **I**, **L**, and **O**), EphA3 (red; **J**, **M**, and **P**), and a merged composite (**K**, **N**, and **Q**) in normal (**I**–**K**) and IPF (**L**–**Q**) lung explants. *n* = 5–7/group. White arrowheads highlight cells where colocalization is observed. (**R**–**V**) Representative IHC images stained for CCR10 (red) and EphA3 (brown) in normal lung (**R**), IPF lung biopsies (**S** and **T**), and IPF lung explants (**U** and **V**) taken at original magnification 50× (top) and 200× (bottom). The respective IgG isotype control staining is shown in the inlaid images. *n* = 5–8/group.

We recently identified a subset of CCR10^+^ epithelial cells in IPF lungs that expressed significantly higher levels of Eph receptor A3 (EphA3) protein compared with CCR10^–^ IPF and CCR10^+/–^ normal epithelial cells (39). Since EphA3 is a putative marker for mesenchymal cells (44), we hypothesized that EphA3 was also differentially expressed in mesenchymal cells from patients with IPF. To further address this, we used Ingenuity Pathway Analysis (QIAGEN) to analyze microarrays of IPF and normal lung samples to generate a list of tyrosine kinases that have been implicated/suspected in fibrosis but are not known targets of nintedanib. From this grouping *EPHA3* transcript was consistently expressed at the highest levels in IPF lung samples compared with the other tyrosine kinases (Supplemental Table 2). A significant elevation was seen in the percentage of EphA3^+^ cells and GMFI of EphA3 in cultured IPF fibroblasts compared with normal fibroblasts (Figure 2, A–C). Increased percentage of EphA3^+^ cells and EphA3 GMFI within PDGFRα^+^ cells (Figure 2, D–F), as well as total CCR10^+^EphA3^+^ cells (Figure 2, G and H) and CCR10^+^ cells within EphA3^+^ cells, were observed in IPF fibroblasts. Although CCR10^+^EphA3^+^ cells were rarely detected in normal lung samples (Figure 2, I–K, arrowheads), these cells were abundant in IPF lung samples (Figure 2, L–Q, arrowheads). In addition, using dual-color IHC analysis, we observed that CCR10^+^EphA3^+^ cells were rarely localized in normal lung explants (Figure 2R) while various CCR10^+^EphA3^+^ cell types were clearly visible in IPF lung biopsies (Figure 2, S and T) and explants (Figure 2, U and V). Together, these results demonstrate that CCR10^+^ fibroblasts and other cell types coexpress EphA3, and these cells are more abundant in IPF compared with normal human lungs.

### EphA3 ligand activates human lung fibroblasts.

We next determined the effect of EphA3 ligand on lung fibroblast/progenitor cell activation. EphA3 was abundantly expressed in cultured IPF lung fibroblasts (Supplemental Figure 5A), and the addition of preclustered Ephrin A5–Fc (EFNA5-Fc) to these cells strongly promoted the phosphorylated EphA3 receptor, indicating the activation of this receptor (Supplemental Figure 5B). Further, preclustered EFNA5-Fc altered soluble collagen type I (Supplemental Figure 5C) and significantly increased IL-6 levels (Supplemental Figure 5E). However, there were no significant changes in αSMA protein levels in the stimulated cells (Supplemental Figure 5D). Last, both normal and IPF SSEA4^+^ mesenchymal progenitor cells (MPCs) expressed cell surface EphA3 protein (Supplemental Figure 5F). These results suggest that MPCs and their fibroblast progeny express EphA3 and suggest the activation of this receptor via EFNA5 induced IL-6 generation.

### IPF and normal MPCs express CCR10, and CCL28 promotes their proliferation.

**Figure 3:**
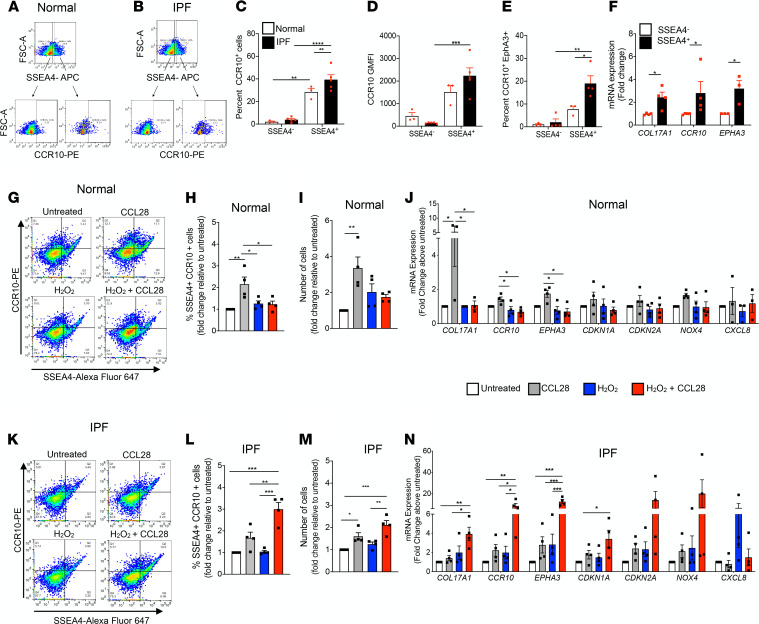
IPF SSEA4^+^ progenitor cells highly express CCR10 protein and CCL28 promotes their expansion. (**A** and **B**) Representative flow cytometry dot plots showing CCR10^+^ (PE) cells within SSEA4^–^and SSEA4^+^ (APC) cells from normal (**A**) and IPF (**B**) lung fibroblasts. The average percentage of CCR10^+^ cells within gated SSEA4^+^ cells (**C**) and average GMFI for CCR10 (**D**) in normal and IPF SSEA4^+^ versus SSEA4^–^ cells are depicted. Average percentage of CCR10^+^EphA3^+^ cells within gated SSEA4^+^ cells (**E**). Transcript expression in SSEA4^+^ versus SSEA4^–^ IPF cells (**F**). (**G–N**) Magnetically enriched SSEA4^+^ lung fibroblasts were treated with CCL28 (200 ng/mL) and/or hydrogen peroxide (H_2_O_2_; 5 μM) for 7 days. Representative flow cytometric dot plots (**G** and **K**) and respective fold change in the percentage of SSEA4^+^CCR10^+^ (**H** and **L**), total number of cells (**I** and **M**), and transcript expression (**J** and **N**) in normal and IPF lung fibroblasts, respectively. Data shown are mean ± SEM; *n* = 3–5/group. *P ≤ 0.05, **P ≤ 0.01, or ***P ≤ 0.005 via 1-way ANOVA test with Tukey’s multiple comparisons test. *CCR10*, CC chemokine receptor 10; *CDKN1A*; cyclin-dependent kinase inhibitor 1A; *COL17A1*, collagen type XVII alpha 1 chain; *CXCL8*, CXC motif chemokine ligand 8; *EPHA3*, Eph receptor A3; *NOX4*, NADPH oxidase 4.

SSEA4^+^ MPCs have been shown to be a major cell of origin for fibroblasts (SSEA4^–^) comprising the fibrotic reticulum in IPF (45, 46). In the present study, we detected SSEA4^+^ progenitor cells in both normal and IPF lung fibroblasts that coexpressed CCR10 (Figure 3, A–C). A significant increase in the percentage of CCR10^+^ cells was observed within IPF SSEA4^+^ MPCs compared with their SSEA4^–^ counterparts and normal SSEA4^+^ MPCs (Figure 3C). IPF MPCs also expressed higher levels of cell surface CCR10 protein compared with IPF SSEA4^–^ fibroblasts (Figure 3D). Interestingly, CCR10^+^ MPCs from normal and IPF lungs coexpressed EphA3, and the percentage of CCR10^+^EphA3^+^ cells was significantly higher in IPF MPC population compared with normal MPCs and IPF SSEA4^–^ fibroblasts (Figure 3E). Analysis of IPF SSEA4^+^ versus SSEA4^–^ cells revealed higher levels of *COL17A1*, *CCR10*, and *EPHA3* transcripts in SSEA4^+^ cells (Figure 3F), which have been shown to be enriched in SSEA4^+^ MPCs (47) and epidermal stem cells (48). Together, these results suggested that CCR10 and *EPHA3* transcripts and protein expression are higher in IPF MPCs compared with MPCs from normal donor lungs.

To investigate the role of CCL28/CCR10 pathway in MPCs, SSEA4^+^ cells were isolated from fibroblast cultures, treated with CCL28 and/or hydrogen peroxide (a preconditioning growth and survival stimulus for mesenchymal/multipotent stromal cells) (49), and analyzed 7 days later. CCL28 increased the percentage of SSEA4^+^CCR10^+^ cells (Figure 3, G, H, K, and L), total cell numbers (Figure 3, I and M), and *CCR10*, *COL17A1*, and *EPHA3* transcripts (Figure 3, J and N) in cultures of normal and IPF MPCs. Moreover, a notable increase in the expression of senescence markers *CDKN1A* (*P* < 0.05), *CDKN2A* (NS), and *NOX4* (NS) was also observed in CCL28- and hydrogen peroxide–treated cultures of IPF MPCs (Figure 3N). Interestingly, hydrogen peroxide significantly increased CCL28-mediated effects and *CXCL8* expression in IPF but not normal MPC cultures (Figure 3, G–N), suggesting that CCR10 ligand promotes the expansion of normal and IPF CCR10^+^ MPCs and that hydrogen peroxide enhances this effect in IPF, but not normal, MPC cultures.

### CRISPR/Cas9-mediated KO of CCR10 results in the loss of SSEA4^+^ MPCs.

**Figure 4:**
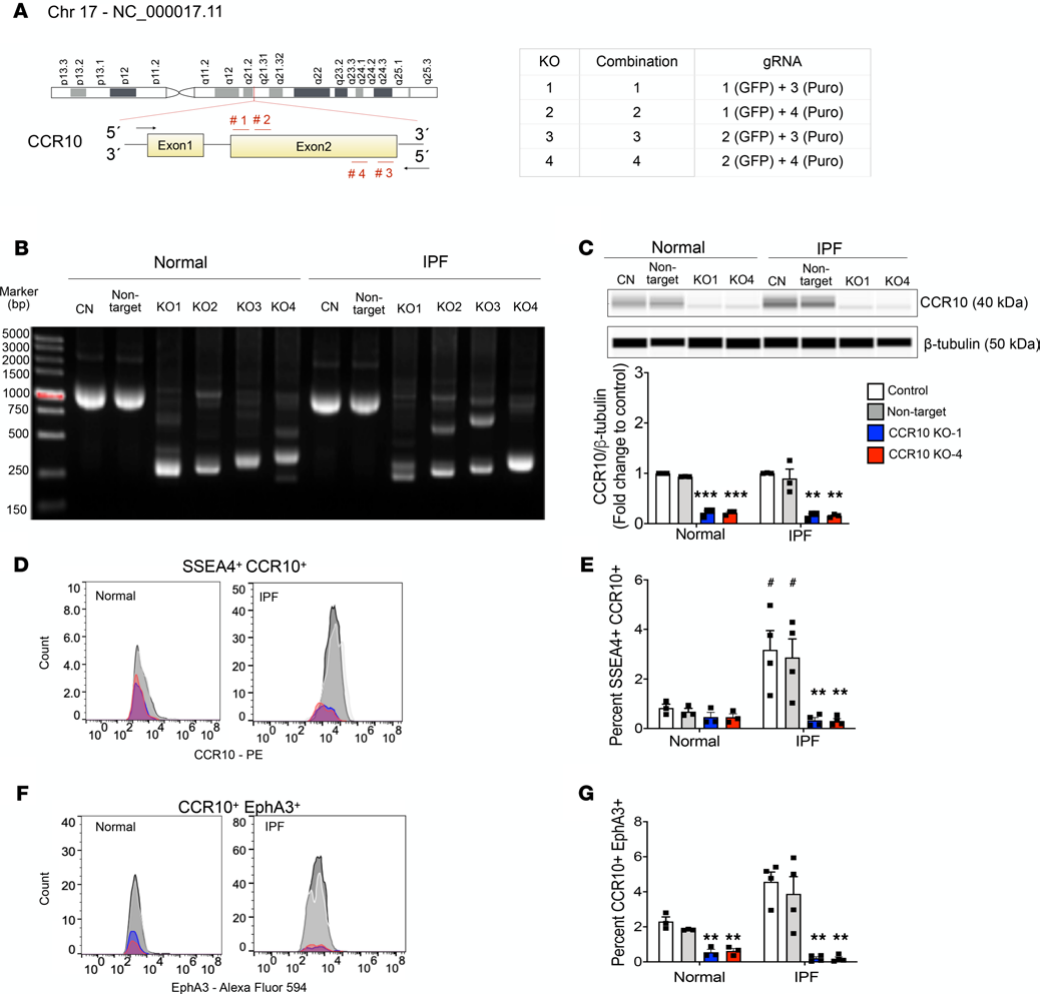
CRISPR/Cas9–mediated KO of CCR10 decreases SSEA4^+^ MPCs. (**A**) Schematic representation of the combinations of gRNAs #1–4 targeting exon 2 of CCR10 in normal and IPF fibroblasts. Normal and IPF fibroblasts were infected with indicated virus, and puromycin-expressing (Puro^+^) cells were first selected with puromycin; then GFP^+^ cells among the Puro^+^ cells were selected by FACS. Sorted GFP^+^Puro^+^ cells are CCR10-KO cells, and empty plasmids were used as negative control (nontarget). (**B**) CCR10-KO validation at genome level. (**C**) Representative Western blot and relative CCR10/β-tubulin expression normalized to respective control and (**D** and **F**) flow cytometric histograms and respective percentages of SSEA4^+^CCR10^+^ (**E**) and CCR10^+^EphA3^+^ cells (**G**). Full uncut gel shown in online supplemental material. Data shown are mean ± SEM; *n* = 3–4 fibroblast lines/group. **P ≤ 0.01, ***P ≤ 0.001 compared with respective control and nontarget; ^#^*P* ≤ 0.05 compared with control and nontarget normal via 1-way ANOVA test with Tukey’s multiple comparisons test.

**Figure 5:**
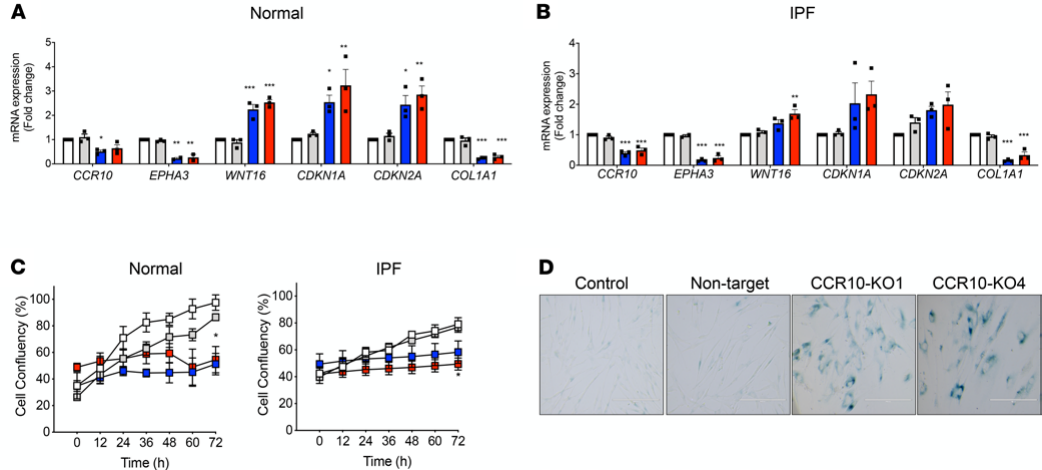
CRISPR/Cas9–mediated KO of CCR10 decreases MPC markers and premature senescence of the fibroblast progeny. Normal and IPF fibroblasts were infected, and Puro^+^ cells were first selected with puromycin; then GFP^+^ cells among the Puro^+^ cells were selected by FACS. Sorted GFP^+^Puro^+^ cells are CCR10-KO cells, and empty plasmids were used as negative control (nontarget). Transcript levels in normal (**A**) and IPF (**B**) control (white), nontarget (gray), and CCR10 KO-1 (blue) and -4 (red) fibroblasts. (**C**–**D**) Control, nontarget, and CCR10 KO-1 and -4 were plated, and proliferation and senescence-associated β-galactosidase expression were assessed. (**C**) Shown is percentage confluence in normal and IPF fibroblasts. (**D**) Representative senescence-associated β-galactosidase expression in IPF fibroblast cultures. Original magnification, 20×. Data shown are mean ± SEM; *n* = 3–4 fibroblast lines/group. (**A**–**C**) *P ≤ 0.05, **P ≤ 0.01, ***P ≤ 0.001 compared with control via 1-way ANOVA test (**A** and **B**) or via 2-way ANOVA test (**C**) with Tukey’s multiple comparisons test.

To further study the role of CCR10 in MPCs and fibroblasts, CRISPR/Cas9 gene editing system was employed to generate CCR10-KO cell lines. Normal and IPF fibroblast cultures were transduced with combinations of 4 guide RNAs (gRNAs) (combinations 1–4; Figure 4A), and deletion of the CCR10-targeted sequence was confirmed by PCR product bands at 294 bp compared with the control (WT and nontargeting control) bands at 1325 bp (Figure 4B). Combinations 1 (i.e., KO-1) and 4 (i.e., KO-4) were selected for further CRISPR/Cas9–mediated KO validation. Western blot and flow cytometry analysis confirmed that CCR10 protein expression (Figure 4C and Supplemental Figure 6A) and CCR10^+^ cells (Supplemental Figure 6, B and C) were markedly reduced compared with the controls. CCR10-KO fibroblast cultures also exhibited a significant reduction in the percentage of SSEA4^+^CCR10^+^ (Figure 4, D and E) and CCR10^+^EphA3^+^ cells (Figure 4, F and G) in normal and IPF cultures. In agreement with these observations, *CCR10* and *EPHA3* transcripts were also reduced in CCR10-KO cultures (Figure 5, A and B). Increased expression of senescence-associated transcripts *WNT16*, *CDKN1A*, and *CDKN2A* and decreased *COL1A1* expression were observed (Figure 5, A and B). Concomitantly, cultured normal and IPF CCR10 KO-1 and KO-4 fibroblasts exhibited reduced proliferation (Figure 5C), as well as the expression of senescence-associated β-galactosidase (Figure 5D), compared with control cultures. Together, these data suggest that CRISPR/Cas9–mediated targeting of CCR10 results in the loss of MPCs and premature senescence of the fibroblast progeny.

### Human CCR10^+^ cells promote fibrosis in NOD/SCID-γ mice following intravenous injection.

**Figure 6:**
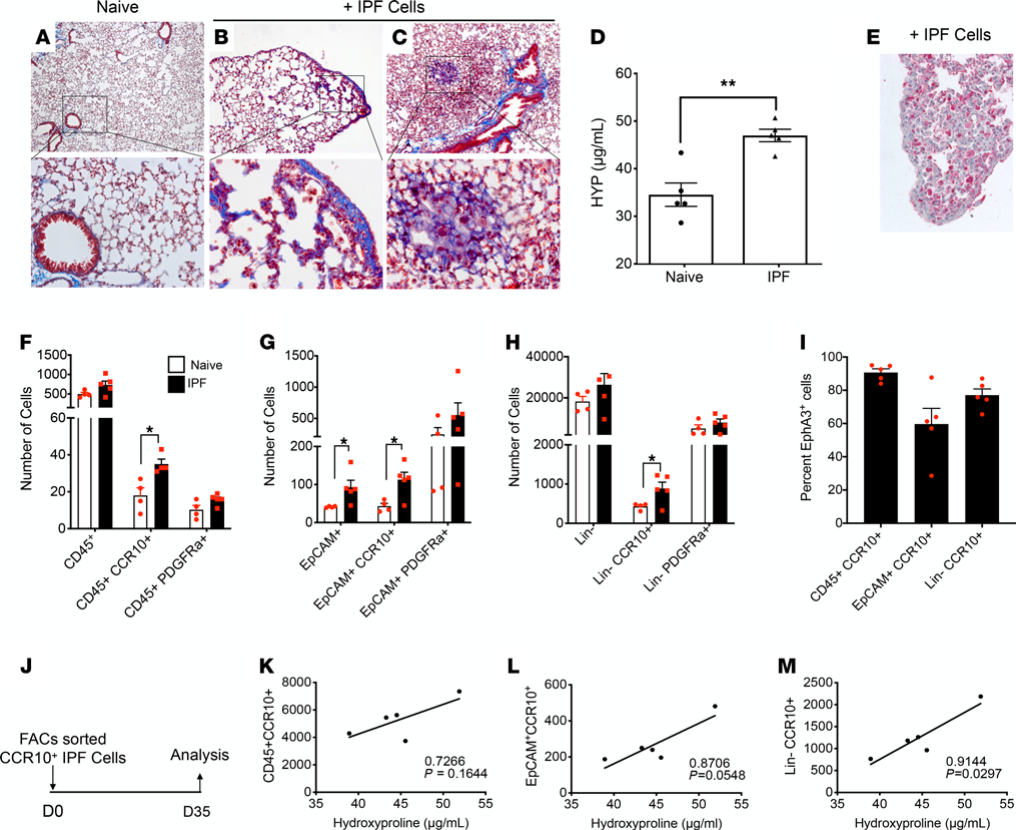
IPF lung cells promote fibrosis in the lungs of NSG mice. (**A**–**C**) Representative images of Masson’s trichrome staining of nonhumanized NSG lung (**A**) and humanized NSG lungs (**B** and **C**) at day 63 after intravenous injection of IPF cells. Images were acquired at original magnification 50× (top) and 200× (bottom). Blue color depicts stained collagenous regions. *n* = 5/group. (**D**) Hydroxyproline in humanized and nonhumanized NSG lungs. **P < 0.01 via 2-tailed Mann-Whitney nonparametric test. (**E**) Sixty-three days after IPF cell injection, lungs from NSG-GFP mice were stained in red for GFP protein. Representative image showing both the GFP^+^ cells (i.e., mouse) and the GFP^–^ cells (i.e., introduced human) is shown at original magnification 200×. (**F**–**H**) NSG-GFP lungs were analyzed by flow cytometry. Shown is the average number of GFP^–^ cells, expressing human CD45, CCR10, EpCAM, and/or PDGFRα proteins in humanized (IPF; black) relative to nonhumanized mice (naive; white). (**I**) Shown is the average percentage of CD45^+^CCR10^+^ and Lin^–^CCR10^+^ expressing EphA3. Data shown are mean ± SEM; *n* = 4–5/group. *P ≤ 0.05 via 2-tailed Mann-Whitney nonparametric test. (**J**–**M**) CCR10^+^ IPF lung cells were sorted and injected intravenously into NSG mice (**J**), and depicted are correlation analyses of hydroxyproline and number of human CD45^+^CCR10^+^ (**K**), EpCAM^+^CCR10^+^ (**L**), and Lin^–^CCR10^+^ (**M**) cells in NSG lungs at day 35 after cell injection. *n* = 5/group. Pearson’s correlation coefficient with *P* values indicated.

Compared with naive (i.e., nonhumanized) NOD/SCID-γ (NSG) mice (Figure 6A) fibrosis was evident histologically in the lungs of humanized mice (Figure 6, B and C) at day 63 after intravenous injection of IPF cells. Further, hydroxyproline was significantly elevated in humanized NSG mouse lungs compared with nonhumanized NSG lungs (Figure 6D). To track the xenografted human cells, cells were administered into NSG mice that were transgenic for EGFP (NSG-GFP). Sixty-three days after IPF cell injection into NSG-GFP mice, GFP^–^ cells were present in NSG-GFP lungs (Figure 6E). Relative to nonhumanized NSG-GFP mice, GFP^–^CD45^+^CCR10^+^, GFP^–^EpCAM^+^CCR10^+^, and GFP^–^Lin^–^CCR10^+^ cells were significantly increased in the humanized mice (Figure 6, F–H), and the majority of the CD45^+^CCR10^+^ and Lin^–^CR10^+^ cells coexpressed EphA3 (Figure 6I). In separate experiments, FACS-sorted CCR10^+^ IPF cells were intravenously administered into NSG mice (Figure 6, J–M), and at day 35 after injection, the numbers of CD45^+^CCR10^+^ (Figure 6K), EpCAM^+^CCR10^+^ (Figure 6L), and Lin^–^CCR10^+^ (Figure 6M) cells were found to positively correlate with hydroxyproline levels in the lungs of humanized NSG mice. Together, these findings demonstrate that CCR10^+^ IPF cells engraft in NSG and NSG-GFP mice, and the presence of these cells positively correlated with lung fibrosis in these mice.

### Normal lung cells do not promote interstitial lung remodeling in humanized NSG mice.

Given our finding that IPF Lin^–^CCR10^+^ cells induced disease in NSG mice, we next assessed whether equivalent cells cultured from normal lung samples promoted lung remodeling in this model. We have previously reported that IPF lung fibroblasts (devoid of CD45 and EpCAM (50, 51) induce lung remodeling in SCID-beige mice, but normal lung fibroblasts do not induce any lung remodeling in SCID-beige mice at day 63 after injection (37). In the present study, we confirmed these previously published findings that normal lung fibroblasts do not cause remodeling in the lungs of immunodeficient mice (Supplemental Figure 7A). We also assessed the effects of mixed normal donor lung cells in humanized NSG mice, which received 1 × 10^6^ normal lung or IPF cells by intravenous injection. At day 63 after injection, there was a significant increase in interstitial collagen in the lungs of NSG mice that received IPF, but not normal, lung cells compared with naive NSG lungs (Supplemental Figure 7, B–E). Together, these results confirm that IPF, but not normal, lung-derived cells promote lung fibrosis in NSG mice.

### Targeting CCR10^+^EphA3^+^ cells ameliorated lung fibrosis in humanized NSG mice.

**Figure 7:**
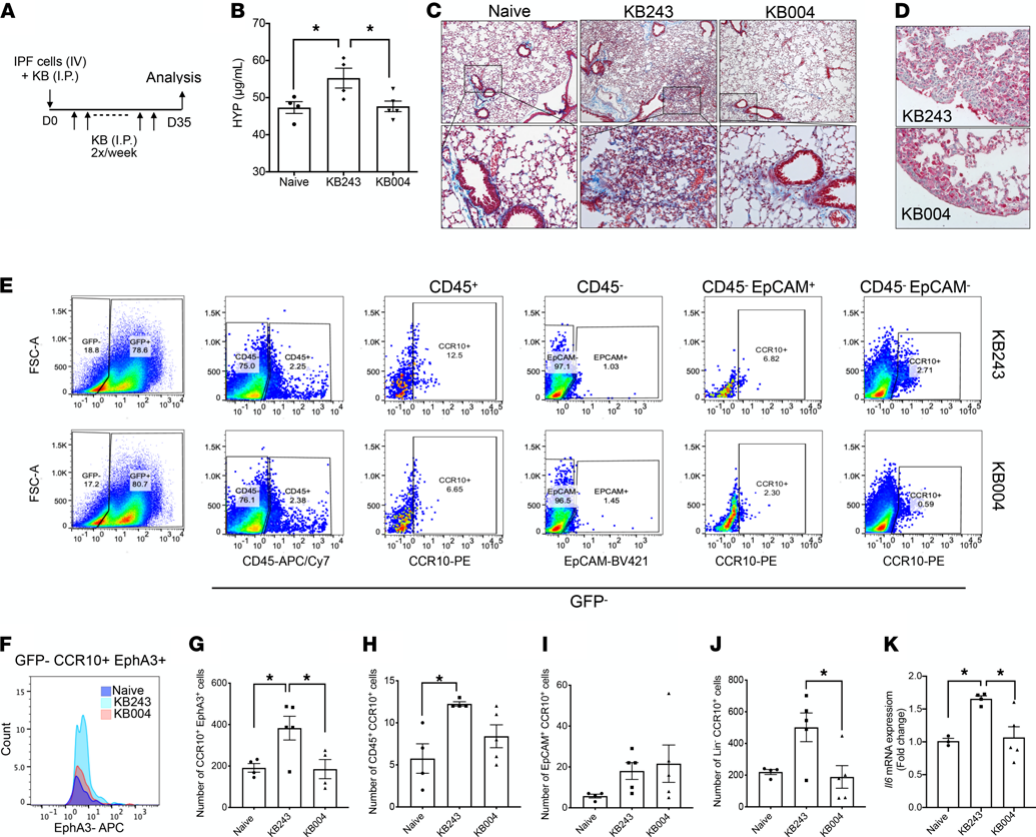
Targeting of CCR10^+^EphA3^+^ cells prevented the development of fibrosis in humanized mice. (**A**) Experimental scheme: on day 0, NSG or NSG-GFP mice received intravenous injection of IPF cells and were treated twice a week with either 5 mg/kg of KB004 (an afucosylated anti-EphA3 mAb) or KB243 (an isotype control) via intraperitoneal injection for 5 weeks. (**B**) Hydroxyproline in nonhumanized NSG mice and humanized NSG mice that received either KB243 or KB004. Data shown are mean ± SEM; *n* = 4–5/group; *P ≤ 0.05 compared with control via 2-way ANOVA test with Tukey’s multiple comparisons test. (**C**) Representative images of Masson’s trichrome–stained lungs from nonhumanized, saline-treated NSG lungs and humanized NSG mice at day 35 after IPF cell injection and treatment with either KB243 or KB004. Shown are images taken at original magnification 50× (top) and 200× (bottom). (**D**) Depicted are representative images of GFP-stained NSG mouse lung (red indicates the presence of transgenic GFP^+^ mouse cells) at day 35 after IPF lung-derived cell injection in both the KB243 and KB004 treatment groups. (**E**–**J**) Flow cytometric analysis of cells in the lungs of nonhumanized and humanized NSG-GFP mice treated with either KB243 or KB004 antibodies. Representative dot plots depicting GFP^–^ cells staining for (from left to right) human CD45, CCR10, and/or EpCAM (**E**). Representative flow cytometric histogram (**F**) and respective average number of GFP^–^CCR10^+^EphA3^+^ cells (**G**). Average number of GFP^–^CD45^+^CCR10^+^ (**H**), GFP^–^EpCAM^+^CCR10^+^ (**I**), and GFP^–^Lin^–^CCR10^+^ cells (**J**). Fold change in the levels of mouse *Il6* mRNA (**K**). Data shown are mean ± SEM; *n* = 4–5/group (**A**–**J**) and *n* = 2–4 (**K**). *P* value indicated or *P ≤ 0.05 via 1-way ANOVA test with Tukey’s multiple comparisons test.

To address the profibrotic effects of CCR10^+^EphA3^+^ cells in vivo, humanized NSG mice were treated with 5 mg/kg of either anti-EphA3 mAb ifabotuzumab (KB004) or IgG isotype control (KB243) mAb (52, 53), as shown in Figure 7A. Hydroxyproline was significantly increased in the lungs of humanized NSG mice treated with KB243; however, KB004 treatment significantly reduced hydroxyproline to levels observed in naive nonhumanized NSG lungs (Figure 7B). Interstitial remodeling and collagen staining were observed in the KB243-treated group (Figure 7C) but not in the KB004-treated group, which appeared similar to lung samples from naive nonhumanized mice. To confirm human cell targeting, antibody treatment was administered in humanized NSG-GFP mice (days 0–35) (Figure 7, D–K). IHC analysis for GFP^–^ cells at day 35 after IPF cell injection showed fewer GFP^–^ cells in KB004- compared with KB243-treated (Figure 7D) humanized NSG-GFP mice. Confirming human cell targeting, KB004 treatment significantly reduced the total number of CCR10^+^EphA3^+^ (Figure 7, E–G), as well as Lin^–^CCR10^+^ cells (Figure 7J), compared with the KB243 treatment group. No effect of KB004 was observed on CD45^+^CCR10^+^ or EpCAM^+^CCR10^+^ cells compared with KB243 (Figure 7, H and I). Neither human IL-6 transcript nor protein was detected in the mouse lungs. Mouse IL-6 transcript levels were significantly increased in xenografted mice compared with naive, which were mitigated by KB004 treatment (Figure 7K).

To determine the therapeutic effects of KB004, separate groups of NSG mice were treated with either KB243 or KB004 starting at day 35 after IPF cell administration (Supplemental Figure 8A). Hydroxyproline was significantly elevated in humanized KB243-treated NSG lungs relative to nonhumanized mice (Supplemental Figure 8B). However, while a subset of KB004-treated NSG mice showed a reduction in hydroxyproline levels relative to KB243-treated groups (Supplemental Figure 8B) and a significant reduction in the number of CD45^+^CCR10^+^ cells (Supplemental Figure 8, D and E), there was no significant difference in lung hydroxyproline content (Supplemental Figure 8B), histological appearance (Supplemental Figure 8C), EpCAM^+^CCR10^+^ or Lin^–^CCR10^+^ cell numbers, or mouse IL-6 transcript levels (Supplemental Figure 8, D and F–H) between KB243- and KB004-treated groups.

One potential explanation for the lack of a therapeutic effect of KB004 in this model pertains, in part, to the diminished numbers of human immune effector cells over time, required for KB004-mediated antibody-dependent cellular cytotoxicity (ADCC) of CCR10^+^EphA3^+^ cells in this model. Thus, a separate group of NSG mice were administered KB243 or KB004 beginning at day 7 after IPF cell injection (Supplemental Figure 9A). Lung hydroxyproline was significantly reduced (Supplemental Figure 9B), histological interstitial consolidation was ameliorated (Supplemental Figure 9C), and there was a significant reduction in the number of CD45^+^CCR10^+^ but not EpCAM^+^CCR10^+^ or Lin^–^CCR10^+^ cells (Supplemental Figure 9, D–G) in KB004- compared with KB243-treated groups. Last, mouse IL-6 transcript levels were also reduced in the lungs of KB004-treated mice (Supplemental Figure 9H).

We noted that neither KB243 nor KB004 treatments in humanized NSG mice evoked inflammation in the mouse lung based upon findings from a Bioplex analysis for murine IL-2, IL-10, IL-12-p70, and TNF-α (Supplemental Figure 10, A–E) in the bronchoalveolar lavage (BAL) from naive and humanized mice. These results demonstrate that KB004-directed targeting of CCR10^+^ IPF cells in humanized NSG mice was less efficacious therapeutically compared with its effects preventatively, possibly due to the loss of immune effector cells with time in this NSG mouse model of lung fibrosis.

## Discussion

The identity of fibrotic triggers in IPF remains elusive, but it is speculated that persistent lung injury might lead to activation of lung fibroblasts. Herein, we characterized immune and nonimmune cells that express CCR10. CCR10, or GPR2, is expressed on various normal and neoplastic cell types (54–57), and it binds CCL27 (58) and CCL28 (59). The transcript and protein expression of this receptor was highest in IPF lungs from patients who experienced rapid progression (as defined previously, ref. 60) over the first year after diagnosis compared with IPF patients showing slow or stable disease and normal donors. Further, this chemokine receptor was abundantly expressed on IPF fibroblasts and SSEA4^+^ MPCs. Intravenous introduction of IPF but not normal donor lung cells into NSG mice promoted fibrosis; most importantly, CCR10^+^ cells coexpressed the receptor tyrosine kinase EphA3; and targeting CCR10^+^EphA3^+^ cells prevented or eliminated fibrosis in NSG mice. Together, these findings demonstrate that cells from patients with IPF expressing CCR10 and EphA3 might be profibrotic.

The altered expression of CCR10 and its ligand CCL28 in IPF was an unexpected finding, given the preponderance of the literature describing these chemokine factors outside the lung. CCL28 was expressed in AT2 cells and CD68^+^ immune cells in normal and IPF lungs. However, CCL28 secretion was increased in basal like cells and senescent fibroblasts from IPF lungs, and CCL28 transcript levels in peripheral blood mononuclear cells from IPF patients correlated with reduced progression-free survival, which is in line with the observed expression of CCR10 in rapidly progressive IPF patients. Together, these findings demonstrate that the expression of and/or the number of cells expressing CCR10 and its ligand CCL28 are increased in IPF, and their abundance appears to be tied to the progressive nature of this disease.

In vitro characterization of cultured IPF Lin^–^ cells showed that among the chemokine receptors assessed, CCR10 was most abundantly expressed. Surprisingly, although CCL28 increased soluble collagen 1 secretion in normal and IPF fibroblasts, no significant effect on other markers of fibroblast activation was observed. Therefore, we postulated that CCR10 may have another role in this population of cells. Further characterization revealed that normal and IPF SSEA4^+^ cells, previously described as an MPC and the source of fibroblasts in the IPF lung (46), expressed significantly more cell surface CCR10 than SSEA4^–^ cells (i.e., fibroblast progeny). CCL28 induced *CCR10*, *COL17A1*, and *EPHA3* transcripts and promoted the proliferation of CCR10^+^SSEA4^+^ MPCs and generation of fibroblast progeny, which is in line with a previous report identifying CCL28 as a growth and survival factor for hematopoietic progenitor cells (61). Interestingly, CCL28-mediated effects were enhanced by hydrogen peroxide in IPF but not in normal cells. This effect may be secondary to hydrogen peroxide–induced *CXCL8* (IL-8 transcript) expression in IPF cells, since IL-8 can promote the self-renewal of IPF MPCs and proliferation of progeny (62). In agreement with a role for CCR10 in the maintenance of MPCs, CRISPR/Cas9–mediated KO of CCR10 in normal and IPF fibroblast cultures caused a dramatic decrease in the numbers of CCR10^+^SSEA4^+^ cells, as well as CCR10^+^EphA3^+^ cells. Previous studies have shown that SSEA4^+^ MPCs produce fibroblast progeny manifesting the full spectrum of IPF hallmarks and induce fibrosis upon injection into SCID mice (45, 46, 62). In fact, the loss of SSEA4^+^ MPCs in CCR10-KO cultures resulted in reduced proliferation rates and promoted the upregulation of markers of senescence in the fibroblast progeny. RNA-sequencing analysis of sorted SSEA4^+^ normal and IPF MPCs has revealed that IPF cells are distinct from their normal counterparts (47). These cells are enriched for profibrotic and senescence factors and exhibit global loss of transcripts coding for components of various DNA damage response and repair proteins (47). These intrinsic differences are consistent with the observed interstitial remodeling in IPF but not in NSG mice humanized with normal donor lung cells. Collectively, our results suggest that CCR10-expressing Lin^–^SSEA4^+^ MPCs are abundant in IPF lungs, where these cells contribute to disease progression.

A novel iteration of the humanized mouse model of IPF was recently developed (63) and utilized to elucidate the role of CCR10^+^ cells in IPF. In this model, IPF, but not normal, lung cells induced a significant increase in interstitial collagen deposition in humanized NSG mice. Further, in IPF lung explant cell–humanized mice, hydroxyproline levels were associated with the abundance of human CCR10^+^ cells and significantly correlated with the abundance of Lin^–^CCR10^+^ cells, demonstrating that these cells contribute to fibrosis in the lung. Due to a paucity of validated reagents that target human CCR10 and the fact that the majority of CCR10^+^ cells that engrafted in the lungs of mice coexpressed EphA3, we addressed whether it might be possible to target these cells via a tyrosine kinase receptor–directed approach. EphA3 was detected and strongly expressed in both IPF lung biopsies and explants. Ephrin receptors represent the largest family of receptor tyrosine kinases (reviewed in refs. 64, 65), and signaling via these tyrosine kinases has been shown to be essential for various developmental processes in the embryo. EphA3 protein is expressed by mesenchymal cells during lung development in mice, but it is rarely expressed in normal adult murine lungs (44). In contrast, EphA3 is frequently overexpressed in various malignancies (reviewed in ref. 66), and increased EphA3 has been observed in IPF (67) and multiorgan fibrosis (68). Accordingly, increased percentages of EphA3^+^, EphA3^+^PDGFRα^+^, and EphA3^+^CCR10^+^ cells were observed in IPF fibroblast cultures. IPF fibroblasts also expressed higher levels of EphA3 protein on their cell surface. Treatment of lung fibroblasts with preclustered EFNA5-Fc ligand induced collagen type I and IL-6 protein secretion, which is consistent with the findings of Campbell et al. (69), who demonstrated that EFNA5 activates mouse fibroblasts, and supports our findings that EphA3 contributes to fibrosis in the lung.

To address the role of CCR10^+^EphA3^+^ cells in humanized NSG mice, EphA3-specific, afucosylated mAb (known as KB004 or ifabotuzumab) was used to target EphA3^+^ cells (52, 53). Although we have previously shown that IPF fibroblasts alone mediate nonresolving lung remodeling in the lungs of mice (37, 63), and the number of Lin^–^CCR10^+^ cells positively correlated with hydroxyproline levels in the lungs of mice, KB004 targets EphA3^+^ cells through an ADCC-dependent mechanism, which relies on the presence of immune cells, specifically NK cells (52, 53). Thus, in the present study, mice received a mixture of cells isolated from IPF mechanically dissociated lung explants, including fibroblasts, MPCs, epithelial cells, and immune cells (63). Prophylactic treatment with KB004 but not KB243 over 35 days completely prevented the development of lung fibrosis and increased IL-6 expression. Targeting EphA3^+^ cells significantly reduced CCR10^+^EphA3^+^ as well as Lin^–^ cells expressing CCR10, thereby confirming the expression of EphA3 on CCR10^+^ IPF cells. Conversely, the therapeutic treatment with KB004 from days 35 to 63 failed to reduce the numbers of Lin^–^CCR10^+^ cells and pulmonary fibrosis in humanized NSG mice. A possible explanation for this observation is the reduction of engrafted human effector immune cells (CD45^+^ cells) by KB004 or the induction of ADCC-inhibitory proteins by engrafted profibrotic human cells in this NSG model over time. In fact, when KB004 treatment was implemented earlier, i.e., from days 7 to 35, reduced lung fibrosis and IL-6 expression were observed. There was also a reduction in the number of CD45^+^CCR10^+^ cells, but not EpCAM^+^CCR10^+^ or Lin^–^CCR10^+^ cells, in the lungs of mice. Although it is still not clear to us why collagen accumulation was mitigated in the absence of Lin^–^CCR10^+^ cell depletion, we cannot discard the possibility that the targeting of SSEA4^+^ progenitors that express EphA3 could have reduced collagen production by the fibroblast progeny due to their senescence. We have shown herein that targeting SSEA4^+^CCR10^+^EphA3^+^ progenitors in vitro results in the senescence of IPF fibroblasts, which exhibit reduced *COL1A1* expression.

While we have not observed an inhibitory effect of KB004 on EphA3 activation in cultured fibroblasts and MPCs, the mouse-specific version of KB004 binds and activates EphA3 and subsequently promotes the internalization of receptor-antibody complexes (70–72). To date, we are not aware of studies that have investigated the effect of this antibody over similar periods of time (i.e., approximately >30 days) and whether continued internalization of the receptor might result in overall inhibition of EphA3 function and, thus, decreased collagen deposition. Nevertheless, we cannot affirm that KB004 can modulate EphA3 activation under these circumstances, and we acknowledge the limitations of this approach, as well as the need for future studies addressing these gaps in knowledge.

Studies report KB004 targeting multiple cell types that express EphA3 (52, 54), so we anticipated that KB004 would target EpCAM^+^CCR10^+^ cells as well. However, the number of these cells was not modulated in vivo in any of the treatment regimens. It is likely that the increased expression of the immune checkpoint protein programmed death ligand 1 (PD-L1) by CD45^–^EpCAM^+^ cells from IPF lungs blunted ADCC by NK and/or cytotoxic T cells (73). Engagement of PD-L1 to its receptor, PD1, is known to mediate inhibition of T and NK cell activation (74–76). Given the complex interplay between immune and nonimmune cell populations expressing CCR10 in vivo, future studies will be directed at addressing the effect of these cells separately and combined in the modulation of lung remodeling and repair.

**Figure 8:**
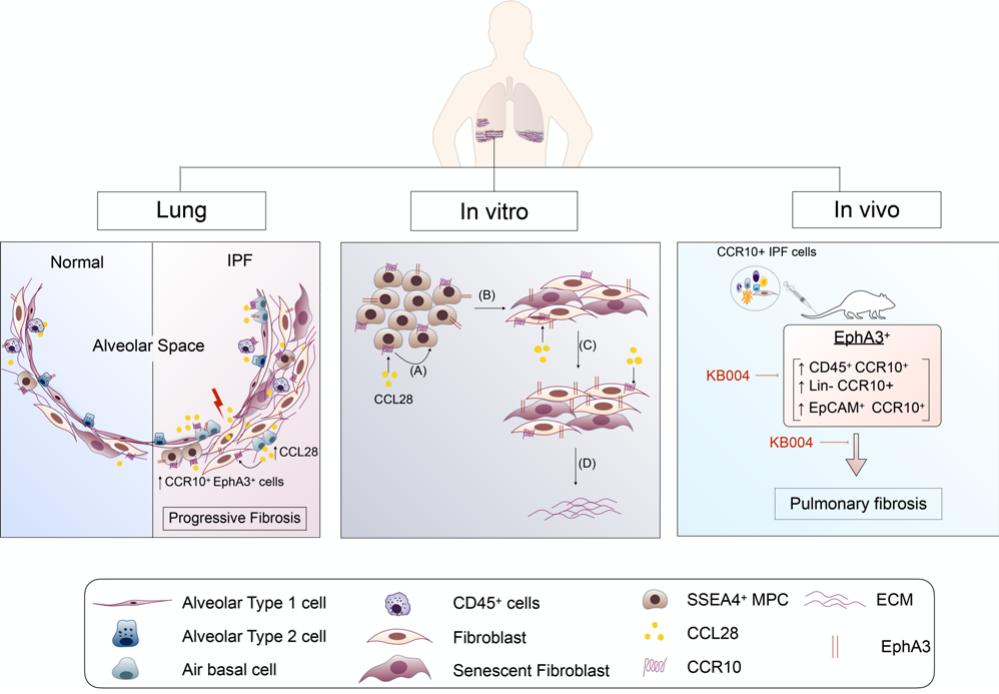
CCR10^+^ cells contribute to progressive fibrosis in IPF. CCR10 and its ligand CCL28 are elevated in the lungs of rapidly progressive IPF patients. CCR10 expression is increased in SSEA4^+^ MPCs from IPF lungs. The receptor tyrosine kinase EphA3 is abundantly expressed on CCR10^+^ cells, and these cells are increased in IPF. CCL28 is secreted by airway basal cells, AT2 cells, and senescent fibroblasts, and, in vitro, it promotes the expansion of CCR10^+^ MPCs (A) and progeny (B), as well as activation [i.e., increased EphA3 expression (C) and collagen secretion (D)] and senescence of their mesenchymal progeny (B). CRISPR/Cas9–mediated KO of CCR10 reduces the percentage of MPCs and CCR10^+^EphA3^+^ cells. Concomitantly, targeting this pathway reduces the proliferative capacity and promotes a senescent phenotype. In vivo, human CCR10^+^ IPF cells initiate and maintain fibrosis in mice. Immune cell–mediated killing of EphA3^+^ cells with ifabotuzumab (KB004) reduces the percentage of CCR10^+^EphA3^+^ cells, as well as CD45^+^CCR10^+^, Lin^–^CCR10^+^, and EpCAM^+^CCR10^+^ cells, and abolishes lung remodeling.

In summary (Figure 8), we have further identified and characterized CCR10 expression by immune, EpCAM^+^, and Lin^–^ cell types, including SSEA4^+^ cells in IPF. In vitro, we demonstrate that CCR10 is required for the activation and proliferation of MPCs. The introduction of CCR10^+^ IPF cells into NSG mice initiated and maintained pulmonary fibrosis in this humanized mouse model of IPF. Human CCR10^+^ cells strongly expressed the receptor tyrosine kinase EphA3, and ADCC-dependent targeting of CCR10^+^EphA3^+^ IPF cells prevented and ameliorated fibrosis in humanized NSG mice. Thus, these findings provide further impetus to explore the targeting of CCR10^+^EphA3^+^ cells in IPF.

## Methods

### Study design

This study was designed to demonstrate the role of CCR10 in IPF and CCR10^+^ cells in promoting pulmonary fibrosis. To this end, we quantified CCR10 and CCL28 transcript and protein expression in peripheral blood and lung biopsies and explants from control and IPF patients exhibiting stable and rapid disease progression, as well as in the various cell types present in these lungs, including structural and immune cells. In vitro, the role of this pathway was studied in mesenchymal cells (i.e., MPCs and fibroblast progeny) by using several complementary approaches, including transcriptomic, flow cytometric, and proteomic analysis, as well as CRISPR/Cas9 genome-editing technique. Next, we injected total or CCR10^+^ FACS-sorted cells from control or IPF explanted lungs into mice, and at day 35 or 63 lung tissue samples were harvested to assess fibrosis and characterization of human cells by flow cytometry. Last, to address the profibrotic effects of CCR10^+^EphA3^+^ cells in vivo, anti-EphA3 mAb ifabotuzumab (KB004) or IgG isotype control (KB243) mAb treatment was administrated from days 0 to 35 or from days 35 to 63 and the aforementioned parameters were evaluated. For all in vivo experiments, mice were age and weight matched across experimental groups, and mice were randomized into naive, humanized, and treatment groups. Operators were not blinded to the treatment during acquisition and analysis of data. Two independent experiments were performed for in vitro and in vivo studies. Study size for experiments was selected on the basis of power calculations of historical data from our laboratory. Sample sizes of *n* = 3 to 6 patient cell lines/group and *n* = 4 to 5 mice/group were used for cell culture experiments and in vivo studies, respectively, as shown in the graphs and described in the figure legends.

### Cells and cell culture conditions

IPF surgical lung biopsy samples were obtained as previously described (60). IPF lung fibroblasts were generated by mechanically dissociating IPF lung biopsies or explants into sterile tissue culture plates as previously described (68). Fibroblasts were cultured in complete media (DMEM; Lonza) containing 15% FBS (Cell Generation), 100 IU of penicillin and 100 mg/mL streptomycin (Lonza), 292 mg/mL l-glutamine (Lonza), and 100 mg/mL Primocin (InvivoGen) at 37°C and 10% CO_2_. For flow cytometric analysis of lung fibroblasts and SSEA4^+^ mesenchymal progenitors, cells were trypsinized for 15–30 seconds, serum-containing medium was added to halt the activity of trypsin, and the cells were spun down at 400*g* for 5 minutes at room temperature. Cells were resuspended at a concentration of 5 million cells/mL, and 100 μL of cells was stained as described in the flow cytometry methods section. For stimulation experiments, 2.5 × 10^5^ cells/well were plated onto a 6-well plate. Cells were incubated overnight prior to stimulations.

### Isolation of mixed cells from IPF explants

Normal and IPF lung explants were acquired from consented donors. IPF progression (i.e., rapid versus slow progression) was defined as previously described (60). Fresh explant cells were isolated and characterized as previously described (63).

### CCL28 correlation analysis

Peripheral blood microarray gene expression data sets from patients with IPF, as part of the COMET IPF cohort (76), were mined for CCL28 transcript expression. The resulting expression values clustered into 3 expression tertiles, and CCL28-low versus -high were defined as lowest expression and the upper 2 expression tertiles, respectively. The expression of CCL28 was then correlated to progression-free survival as defined by death or a 10% decline in FVC. Cox regression analysis was then performed unadjusted or adjusted for SAP score.

### Quantitative PCR analysis

Cells were lysed in TRIzol reagent (Thermo Fisher Scientific), and RNA was extracted and reverse-transcribed into cDNA using SuperScript II Reverse Transcriptase (Life Technologies, Thermo Fisher Scientific) as recommended by the manufacturers and previously described (60). Gene expression analysis was performed using predesigned primers and probes (Thermo Fisher Scientific). All TaqMan analysis was performed using an Applied Biosystems (Thermo Fisher Scientific) ViiA 7 instrument, and fold change values were calculated using Data Assist software (Thermo Fisher Scientific). All human and mouse data were normalized to *RNA18S5* or *GAPDH* expression, respectively.

### Magnetic sorting of CCR10^+^ and SSEA4^+^ cells

Lung fibroblasts were trypsinized and spun down as described above (“Cells and cell culture conditions”). Cells were resuspended at a concentration of 5 × 10^6^ cells/mL and stained with PE-conjugated anti-CCR10 antibodies (BioLegend, 341504) or anti-SSEA4 Microbeads (MACS Miltenyi Biotec, 130-097-855). After staining, cells were washed and then magnetically sorted using anti-PE magnetic beads (STEMCELL Technologies) or LS Columns (Miltenyi Biotec), respectively, as recommended by the manufacturers.

### Stimulation of SSEA4^+^ cells

Magnetically enriched SSEA4^+^ cells were plated overnight (3 × 10^5^ cells/well) onto a 6-well plate and stimulated with CCL28 (200 ng/mL, R&D Systems, Bio-Techne) and/or hydrogen peroxide (5 μM, MilliporeSigma) for 7 days. Fresh conditioned medium was replaced once during this period. On day 7 cells were counted and processed for flow cytometric analysis or quantitative PCR.

### Generation of CCR10-KO cell line with CRISPR/Cas9

Human CCR10 gRNA sequences for CRISPR/Cas9 were designed with the MIT website tool (http://crispr.mit.edu/) (77). The sgRNA sequences used were as follows: #1: 5*′*-GGATGAAGAGGACGCATACT-3*′*; #2: 5*′*-TTGCTACAAGGCCGATGTCC-3*′*; #3: 5*′*-TTCGCAGCCCTAGTTGTCCC-3*′*; #4: 5*′*-CGGCGGGGTTGAGGCCCTGA-3*′*. sgRNA #1 and sgRNA #2 were cloned into LentiCRISPRv2GFP (Addgene plasmid 82416) and sgRNA #3 and sgRNA #4 into LentiCRISPRv2puro (Addgene plasmid 98290) as described (77). The sequences of the sgRNA plasmids were verified by Sanger sequencing. Viruses were generated by cotransfecting sgRNA plasmids with pCMVR8.74 and pMD2G to HEK293T cells (ATCC). Forty-eight hours posttransfection, supernatants were collected, and virus was precipitated with PEG-8000 (Thermo Fisher Scientific: BP233-1). Normal and IPF fibroblasts were infected with indicated virus as shown in Figure 4A overnight, and Puro-expressing cells were first selected with puromycin (2 μg/mL; Life Technologies, Thermo Fisher Scientific) 2 days after initial infection; then GFP^+^ cells among the Puro^+^ cells were selected by FACS. The sorted GFP^+^Puro^+^ cells are CCR10-KO cells, which was validated at genome and protein level via Western blot. To validate CCR10 KO at genome level, nested PCR was conducted, and primer pairs used were as follows: primer set 1: (F) 5*′*-CTCAAGGTCACCCAGGAAGC-3*′* (R) 5*′*-AGGCACAGAGGTAGTCCCTT-3*′*; primer set 2: (F) 5*′*-AGCTGGAAGCAGAGGTAGGA-3*′* (R) 5*′*-CTACTCCCCTTTCCCACGA-3*′*. Empty plasmids were used as negative control (nontarget) for CCR10-KO experiments.

### Flow cytometry

#### Mouse lung cells.

Mouse lung cellular suspensions were generated using a mouse lung dissociation kit, C-tubes, and a GentleMACS dissociator (Miltenyi Biotec). Lung cell suspensions were centrifuged at 300*g* for 5 minutes at 4°C and resuspended in 1 mL of 1× RBC lysis buffer (BioLegend), incubated at room temperature for 1–2 minutes, and then equilibrated by adding 25 mL of Dulbecco’s PBS (DPBS) to each tube. Cells were centrifuged at 300*g* for 5 minutes at 4°C and resuspended in flow cytometric wash/staining buffer (DPBS + 2% FBS) in the presence of human and mouse Fc receptor blocking antibodies (BioLegend). Cells were then stained with anti-human CD45, EpCAM, CCR10, PDGFRα, and/or EphA3 (supplied by Humanigen, Inc.) antibodies (see *Antibodies and data collection and analysis*) for 20 minutes at 4°C. Unstained isotype controls, fluorescence minus one controls, and staining in nonhumanized murine lung suspensions were utilized to gate out any nonspecific antibody binding and background fluorescence. CCR10 antibodies were validated via the enrichment for CCR10 transcripts in magnetically sorted CCR10^+^ cells versus nonsorted normal and IPF explant cells (Supplemental Figure 11).

Representative gating for GFP^–^ cells and xenograft cell gating strategies are summarized in Supplemental Figure 13. Due to the presence of a population of GFP^–^ cells in naive, nonhumanized NSG-GFP mice, all of our comparisons and gates were determined relative to nonhumanized NSG-GFP mice. The resulting gates from nonhumanized NSG-GFP mice were used to identify human-, CD45-, EpCAM-, CCR10-, and PDGFRα-stained cells.

#### Human lung cells from lung explants, fibroblasts, and MPCs.

Human lung fibroblasts or MPCs were added to flow cytometry wash/staining buffer and blocked with anti-human Fc receptor antibodies (BioLegend) for 15 minutes at 4°C. After blocking, cells were stained with anti-human CCR10, CD3, CD4, CD8, CD19, CD68, EpCAM, CD45, EphA3, SSEA4, and PDGFRα for 20 minutes at 4°C. Cells were then washed twice with flow cytometry wash/staining buffer and fixed in 5% neutral buffered formalin (NBF). Representative gating and respective controls of main stainings are summarized in Supplemental Figure 12.

#### Antibodies and data collection and analysis.

We used PE-conjugated anti-human CCR10 (Miltenyi Biotech: 130-104-822 or BioLegend: 341504), BV421- or APC-conjugated anti-human EpCAM (BioLegend: 324220 or 118214, respectively), PE-Cy7 or APC/Fire 750-conjugated anti-human CD45 (BioLegend: 304016 or 368518, respectively), BV421-conjugated anti-human CD19 (BioLegend: 302234 or 363018), APC/Fire 750-conjugated anti-human CD3 (BioLegend: 344840), Percp/Cy5.5-conjugated anti-CD68 (BioLegend: 333814), PE-conjugated anti-human CD4 (BioLegend: 344638), PE/Cy7-conjugated anti-human CD8 (BioLegend: 344712), APC- or Alexa Fluor 594–conjugated anti-EphA3 (supplied by Humanigen, Inc.) or biotin-conjugated anti-human EphA3 (supplied by Humanigen, Inc.) plus FITC-conjugated streptavidin (Thermo Fisher Scientific: SA1001), Alexa Fluor 647–conjugated anti-SSEA4 (BioLegend: 330408) or biotinylated anti-SSEA4 (BioLegend: 330404) plus FITC-conjugated streptavidin (Thermo Fisher Scientific: SA1001), or PE/Cy7-conjugated anti-PDGFRα (BioLegend: 323508). All in vivo flow cytometric data were acquired using a MACSQuant 10 (Miltenyi Biotec) flow cytometer. The in vitro flow cytometric data shown in Figure 1, L–N; Supplemental Figure 1; and Figure 6F were acquired using a MACSQuant 10 (Miltenyi Biotec), whereas data shown in Figures 2–4, Supplemental Figure 6, and Supplemental Figure 12 were acquired using an SA 3800 Spectral Analyzer (Sony) flow cytometer. All data were analyzed using FlowJo software V10.2 (Tree Star Inc.).

### Mice

Female, 6- to 8-week-old, pathogen-free NOD Cg-Prkdc^SCID^ IL2rg^Tm1wil^Szi (NSG) and NOD.Cg-Prkdc^SCID^ IL2rg^tm1Wil^ Tg (CAG-EGFP) 10sb/SzJ (NSG-GFP) were purchased from The Jackson Laboratory and housed in Cedars-Sinai Medical Center’s high-isolation animal rooms. NSG mice were allowed a minimum of 1 week to acclimate in the facility, and then these mice received either nothing (i.e., naive or nonhumanized) or 1 × 10^6^ IPF cells by intravenous injection (i.e., humanized) as previously established (63). In most in vivo experiments, the IPF cells used were removed from liquid N_2_ storage, rapidly thawed, and washed in serum-free medium prior to injection into NSG mice. All humanized mice from the same experiment received cells from the same IPF patient, and each *n* value represents 1 mouse. Randomized humanized NSG mice received KB004 (i.e., anti-EphA3 mAb supplied by Humanigen, Inc.; refs. 52, 53; 5 mg/kg) or KB243 (i.e., appropriate IgG control supplied by Humanigen, Inc.; 5 mg/kg) either from days 0 to 35 or from days 35 to 63 by intraperitoneal injection twice weekly. All NSG groups were monitored daily, and mice were sacrificed and excluded from the study if there was evidence for morbidity, such as weight loss of more than 20%, loss of fur, paralysis, and/or lack of responsiveness when handled. At the indicated times after IPF cell injection, BAL fluid and serum were collected for protein analysis, the superior and middle lobes for biochemical hydroxyproline quantification, the inferior lobe for flow cytometric analysis, the postcaval lobe for quantitative PCR analysis, and the left lung for histological analysis from each NSG mouse.

### Hydroxyproline assay

Total lung hydroxyproline was analyzed as previously described (63). Hydrolyzed lung samples from experimental groups were compared with hydrolyzed lungs from naive, nonhumanized NSG mice that were housed in the same facility under similar conditions.

### Histological analysis

Lung tissue was fixed in 10% NBF solution for 24 hours and subsequently transferred into tissue cassettes and kept in a 70% ethanol solution for approximately 24 hours. Lungs were then processed using routine histology techniques and stained using Masson’s trichrome (Supplemental Methods). A Zeiss Axio Observer Z1 microscope and the Zeiss Zen 2012 v 1.1.2.0 software were used to obtain representative images.

### Statistics

All statistical analyses were performed using GraphPad Prism software version 7 (GraphPad) and described in the respective figure legends. *P* < 0.05 was considered significant.

### Study approval

Institutional Review Boards both at Cedars-Sinai Medical Center and the University of Michigan approved all experiments with primary human tissue. Informed consent was obtained from all patients prior to inclusion in the studies described herein. Cedars-Sinai Medical Center Department of Comparative Medicine and the University of Michigan Unit for Laboratory Animal Medicine approved all mouse studies described herein. Sample size and all studies were determined and performed in accordance with the relevant guidelines and regulations. The demographics for the IPF patients, whose cells were utilized for the studies described in this report, are provided in Supplemental Table 1.

## Author contributions

MSH and DMH conceived, designed, and performed experiments; analyzed results; and wrote and edited the manuscript. CMH conceived and designed the experiments and wrote and edited the manuscript. MSE and GH performed experiments and edited the manuscript. ALC, RN, and IJ performed experiments. AK assisted with experiments. BS, JLM, and GC provided reagents. JMO, IM, and SFM provided CCL28 data sets and performed correlation analysis using results obtained from the IPF COMET study. GY and JW helped conceive experiments and provided reagents for in vivo studies. PWN, DJ, PC, WP, BS, JLM, GC, and LAM edited the manuscript.

## Supplementary Material

Supplemental data

## Figures and Tables

**Table 1:**
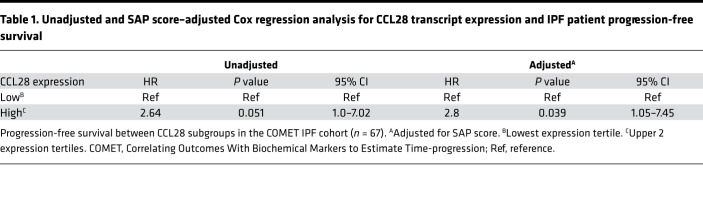
Unadjusted and SAP score–adjusted Cox regression analysis for CCL28 transcript expression and IPF patient progression-free survival

## Version 1 05/04/2021

In-Press Preview

## Version 2 06/08/2021

Electronic publication
