# Supplementary material for: Antibody-mediated depletion of CCR10+EphA3+ cells ameliorates fibrosis in IPF
Source: JCI Insight. 2021 Jun 8;6(11):e141061. doi: 10.1172/jci.insight.141061 (PMC8262321; doi:10.1172/jci.insight.141061)
Supplement: Supplemental data [file jciinsight-6-141061-s046.pdf]

Antibody-mediated depletion of CCR10$^+$ EphA3+ cells ameliorates fibrosis in IPF.

Miriam S. Hohmann[1], David M. Habiel[1], Milena S. Espindola[1], Guanling Huang[1], Isabelle Jones[1], Rohan Narayanan[1], Ana Lucia Coelho[1], Justin M. Oldham[2], Imre Noth[3], Shwu-Fan Ma[3], Adrianne Kurkciyan[1], Jonathan McQualter[1], Gianni Carraro[1], Barry Stripp[1], Peter Chen[1], Dianhua Jiang[1], Paul W. Noble[1], William Parks[1], John Woronicz[4], Geoffrey Yarranton[4], Lynne A. Murray[5], and Cory M. Hogaboam[1*]

Affiliations:

[1]Women's Guild Lung Institute, Division of Pulmonary and Critical Care Medicine, Department of Medicine, Cedars-Sinai Medical Center, Los Angeles, CA, USA, 90048.
[2]Department of Internal Medicine, Division of Pulmonary, Critical Care and Sleep Medicine, University of California at Davis, Sacramento, CA, USA, 95817.
[3]Division of Pulmonary and Critical Care, Department of Medicine, University of Virginia, Charlottesville, VA, USA, 22904.
[4]KaloBios Pharmaceuticals (now Humanigen), Burlingame, CA 94010.
[5]MiroBio Ltd, Winchester House, Heatley Road, Oxford Science Park, Oxford, UK, OX4 4GE.

Online supplemental materials

Supplemental Methods:

Immunohistochemistry

Slides containing 4 µm sections were deparaffinized and hydrated by incubating them in two changes of xylene for five min each, followed by 2 changes of 100% ethanol for 3 min each, 70% ethanol for 2 min, 50% ethanol for 2 min, and distilled water for 5 min. Antigen retrieval was performed by incubating the slides in 10 mM Citric acid solution (pH 6.0) in an 80 ºC oven overnight. The slides were subsequently washed in PBS and permeabilized in 10% methanol containing 0.4% H_2O_2 for 30 min. After permeabilization, slides with murine tissues were washed and stained with a rabbit anti-GFP antibodies and using an aminoethyl carbazole (AEC) substrate kit. IHC analysis on human lung biopsies or explants were performed using a rabbit anti-human CCR10 (Abcam, Ab30718), rabbit anti-CCL28 (Genetex, Inc, GTX108432), rabbit anti-CD68 (Abcam, Ab213363) or rabbit anti-surfactant protein C (Abcam, Ab40879) antibodies. Dual color IHC was performed using a dual color IHC kit (Enzo Lifesciences), rabbit anti-human CCR10 (Abcam, Ab30718) and mouse anti-human EphA3 (Clone, SL2, Humanigen, Inc.) antibodies as recommended by the manufacturer. Immunofluorescence staining was completed by washing the slides following primary antibody incubation and subsequent incubation with fluorescent probe conjugated secondary antibodies for 1 h at room temperature. Slides were then washed and mounted using a DAPI containing mounting medium (Thermo Fisher Scientific). IHC staining for lung fibroblasts was performed as follows: cells were fixed using 4% formaldehyde solution for 10 minutes at room temperature. The cells were then, washed, permeabilized by incubating them in 10% methanol solution for 5 minutes and stained with anti-EphA3 antibodies followed by HRP

conjugated secondary antibodies and DAB developing reagent. All images were acquired using an AxioCam MRc camera Zeiss AX10 microscope using a 5x (0.16 aperture) and 20x (0.8 aperture) lenses (Carl Zeiss Microscopy GmbH) at room temperature and Zen 2012 (Blue edition) v 1.1.2.0 software.

Generation of Senescent Fibroblasts

Senescent fibroblasts were generated as previously described(1). Proliferating NL and IPF lung fibroblasts were serially passaged in culture until the cells showed a senescent phenotype (flattened morphology, permanent growth arrest, and altered gene expression [upregulation of *CDKN1A*, *CDKN2A*, *IL6*, and *IL8* genes]) and senescence-associated b-galactosidase activity (b-Galactosidase Staining Kit; BioVision).

Fibroblast stimulations

Seven-thousand lung fibroblasts per well were plated into a 96 well plate for soluble collagen 1, IL-6, and in cell αSMA and ß-tubulin ELISA analysis or 2.5 x 10^5 cells/well were plated onto a 6-well plate for qPCR analysis. Cells treated with CCL28 (200 ng/mL, R&D systems) for 72 hours (Supplemental Figure 4, B-F); or pre-clustered EFNA5-Fc (100 nM for 72 hours; R&D systems; pre-clustered via incubation with anti-his tag antibodies (R&D systems, MAB05-100) at a 1:10 ratio for 20 minutes at room temperature), IgG, and Ephrin A ligand neutralizing EphA3-FC chimeric protein for 72 hours (Supplemental Figure 5).

Soluble collagen 1, IL-6, and in cell αSMA and ß-tubulin ELISA analysis

Conditioned supernatants were collected for soluble collagen 1 and IL-6 quantification, and the cells were fixed using 4% paraformaldehyde solution in cell αSMA and ß-tubulin ELISA analysis. Commercial ELISA assays (R&D systems) were performed to quantify IL-6 levels as recommended by the manufacturer. Soluble collagen 1 and in cell αSMA and ß-tubulin ELISA assays were carried out as described previously(2).

Western blot

Cells were lysed using cell lysis buffer (Cell Signaling) containing Halt™ Protease and Phosphatase Inhibitor Cocktail (Thermo Fisher Scientific) and briefly sonicated and centrifuged. Protein was quantified from cell lysates using a DC Protein assay (Bio-Rad Laboratories, Inc.). For p-EphA3 quantification, traditional western blot analysis was carried out as previously described(2) using anti-phospho-EphA3 (Cell Signaling, #8862S) and anti-β-tubulin (Abcam, ab6046). CCR10 protein was quantified by capillary western blot using JESS according to the manufacturer's protocol (ProteinSimple, San Jose, CA, USA)(3). Lysed protein samples (1 mg/mL), blocking reagent, wash buffer, anti-CCR10 (Abcam, ab30718) or β-tubulin (Abcam, ab6046), anti-rabbit HRP (R&D systems, HAF008), and chemiluminescent substrate were prepared and loaded into appropriate manufacturer-provided plates. Protein separation and detection was performed automatically on the individual capillaries and analysis was carried out as described by the manufacturer (Figure 4C). All reagents used in this system were provided by ProteinSimple.

Gene expression array data mining and Ingenuity IPA analysis.

To generate a list of tyrosine kinases not targeted by nintedanib, known nintedanib targets(4) were excluded from analysis and the remaining tyrosine kinases were added to a custom Ingenuity pathway generated from the Ingenuity database. Publicly available gene expression datasets (GSE24206) were mined from NCBI's geo datasets database. Groups were defined as follows –IPF lung explants (n=4) vs normal lungs (n=6). Gene expression values were extracted using NCBI's Geo2R gene expression analysis tool and the expression data were uploaded onto ingenuity IPA. Ingenuity IPA was set to only consider changes in gene expression of 1.5-fold or greater and $p \leq 0.05$. Tyrosine kinase expression values were overlaid onto the custom generated list of tyrosine kinases not targeted by nintedanib to generate Table S2.

Fibroblast Proliferation

For fibroblast proliferation, 0.5×10^4 cells control and GFP^+Puro^+ cells (CRISPR-Cas9 mediated CCR10 knockout cells subjected to puromycin selection and FACS sorting for GFP expression) were plated in a 96 well plate (Costar) overnight. Proliferation was assessed every 12 hours over a period of 84 hours at 37 ^oC, 10% CO_2 in the IncuCyte ZOOM live cell imager (Essen Biosciences). The percentage of cell confluence was determined using IncuCyte ZOOM software (Essen Biosciences) as recommended by the manufacturer.

Quantification of interstitial Masson's Trichrome blue staining:

Lung tissues were Masson's trichrome stained and tile images of the stained NSG

lungs were acquired at 10x magnification using a Zeiss AX10 microscope. Images were then stitched and exported using Zen 2012 (blue edition) v 1.1.2.0 software. The exported images were imported into Image pro premier v9.3.3, gamma set to 0.364 and a low pass 2D filter was applied (strength 100, passes 65) to blur the image. Using a manual count-threshold and size exclusion filter, airway and large blood vessel (but not interstitial) Masson's trichrome blue staining was detected (Supplemental Figure 14A). The stained areas were then copied as regions of interest (airway ROI) and superimposed onto the original (non-blurred) version of the same lung. Using a manual count-threshold for interstitial blue staining (Supplemental Figure 14, B-C), interstitial collagen-blue staining was quantified outside the airway ROIs and the total stained areas were then exported. The software was set to quantify total lung tissue outside the airway ROIs (Supplemental Figure 14D). Total area of lung tissue was exported and the ratio of total area of trichrome stained regions by total area of lung tissue was calculated using Microsoft Excel (Microsoft Corporation).

Detection of IL-12p70, IFNγ and TNFα in the BAL from non-humanized and humanized NSG mice.

Lungs in non-humanized and humanized NSG mice were washed using 1 mL of saline solution, and the resulting BAL was centrifuged to remove cells and the supernatants were stored at -80 °C until analysis. Mouse cytokines in the BAL were measured using predesigned Bioplex assays (Bio-Rad Laboratories, Inc.) as recommended by the manufacturer.

Supplemental References:

1. Hohmann MS, Habiel DM, Coelho AL, Verri WA, Jr., and Hogaboam CM. Quercetin Enhances Ligand-induced Apoptosis in Senescent Idiopathic Pulmonary Fibrosis Fibroblasts and Reduces Lung Fibrosis In Vivo. *Am J Respir Cell Mol Biol.* 2019;60(1):28-40.
2. Espindola MS, Habiel DM, Narayanan R, Jones I, Coelho AL, Murray LA, et al. Targeting of TAM Receptors Ameliorates Fibrotic Mechanisms in Idiopathic Pulmonary Fibrosis. *Am J Respir Crit Care Med.* 2018.
3. Harris VM. Protein detection by Simple Western analysis. *Methods Mol Biol.* 2015;1312:465-8.
4. Hilberg F, Roth GJ, Krssak M, Kautschitsch S, Sommergruber W, Tontsch-Grunt U, et al. BIBF 1120: triple angiokinase inhibitor with sustained receptor blockade and good antitumor efficacy. *Cancer Res.* 2008;68(12):4774-82.
5. Guo M, Wang H, Potter SS, Whitsett JA, and Xu Y. SINCERA: A Pipeline for Single-Cell RNA-Seq Profiling Analysis. *PLoS Comput Biol.* 2015;11(11):e1004575.
6. Du Y, Guo M, Whitsett JA, and Xu Y. 'LungGENS': a web-based tool for mapping single-cell gene expression in the developing lung. *Thorax.* 2015;70(11):1092-4.
7. Du Y, Kitzmiller JA, Sridharan A, Perl AK, Bridges JP, Misra RS, et al. Lung Gene Expression Analysis (LGEA): an integrative web portal for comprehensive gene expression data analysis in lung development. *Thorax.* 2017;72(5):481-4.

Supplemental Figures and Legends:

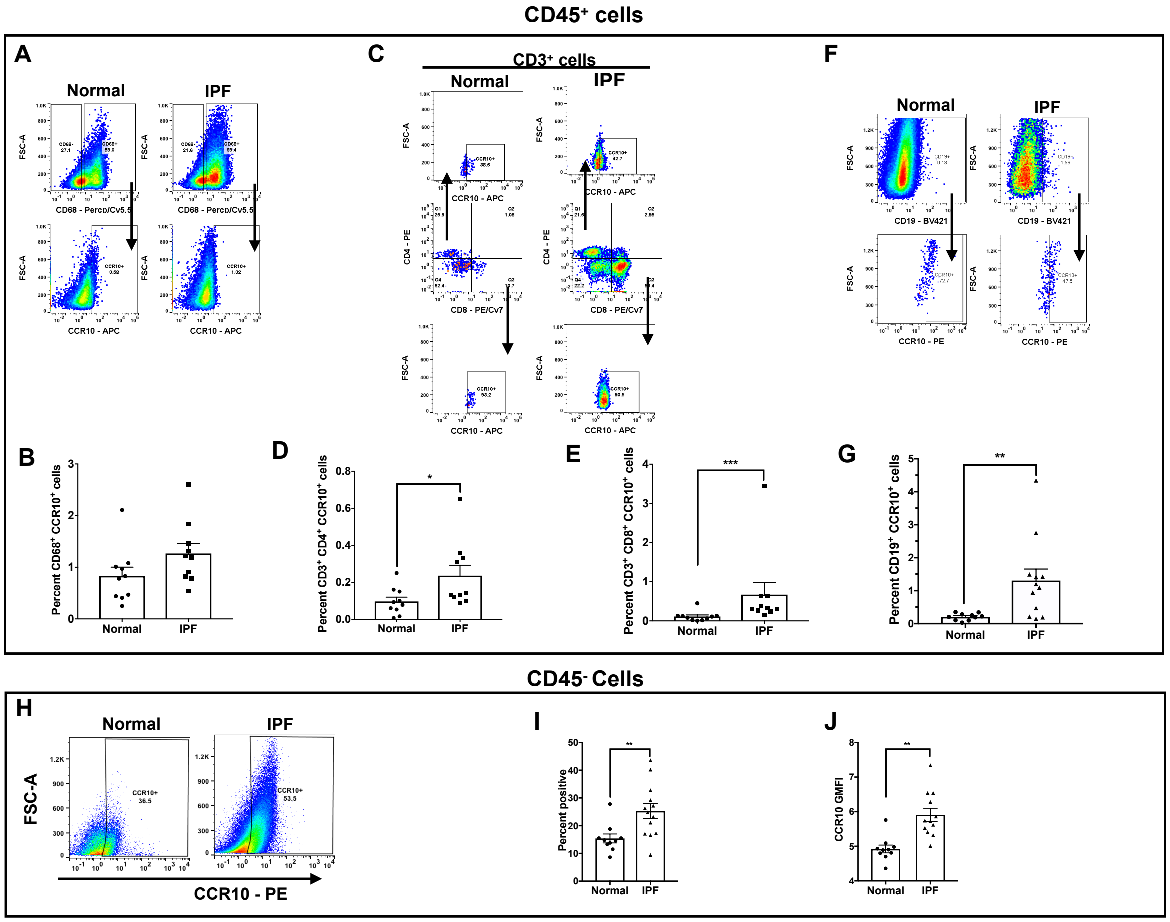

Supplemental Figure 1: Flow cytometric characterization of CD45+ CCR10+ cells in normal and IPF lung explants.

Normal and IPF lung explant cells were stained with anti-CD45, -CCR10, -CD68, -CD3, -CD4, -CD8, and/or -CD19 antibodies and subsequently analyzed by flow cytometry. Depicted are representative flow cytometric dot plots for CCR10 expressing CD68+ (**A**), $CD4^+$, $CD8^+$ (**C**), and $CD19^+$ (**F**) immune cells ($CD45^+$ cells) in normal and IPF lung explants. The average percentage of $CD45^+$ cells expressing $CD68^+$ $CCR10^+$ (**B**), $CD3^+$ $CD4^+$ $CCR10^+$ (**D**), $CD3^+$ $CD8^+$ $CCR10^+$ (**E**), and $CD19^+$ $CCR10^+$ (**G**). (**H**) Depicted are representative flow cytometric dot plots for CCR10 expressing $CD45^-$ cells, (**I**) average

percentage of CCR10^{+} and CCR10 GMFI in normal and IPF explant cells. Data shown are mean ± s.e.m.; n=10-12/group *p ≤ 0.05; **p ≤ 0.01; ***p ≤ 0.001 via two-tailed Mann Whitney non-parametric test.

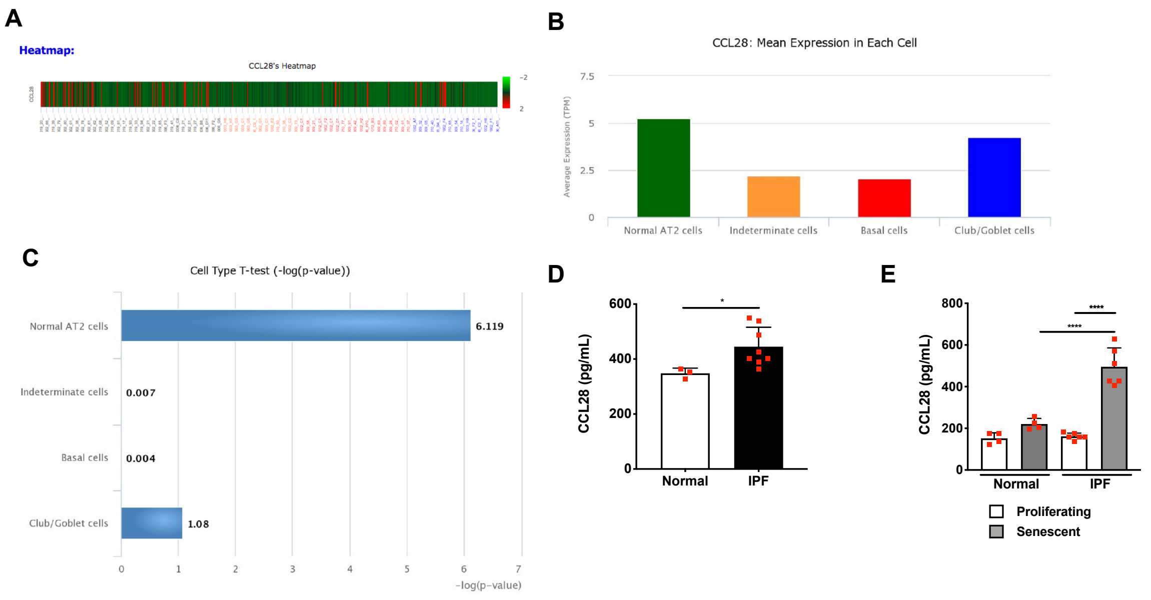

Supplemental Figure 2. CCL28 expression in the lung and tissue derived cultured cells from normal and IPF patients.

(**A-C**) Publicly available single cell RNAseq datasets(5-7) of normal type II alveolar epithelial cells (AT2 cells) and IPF epithelial cells were mined for CCL28 transcript expression. Depicted is a heat map (**A**), average TPM expression (**B**) and the –log(p-value) (**C**) for CCL28 transcript expression in normal and IPF epithelial cells. CCl28 levels in the supernatant of cultured epithelial cell (**D**) from normal (n=3) and IPF (n=8) lung explants. Data shown are mean ± s.e.m.; *p ≤ 0.05 via two-tailed Mann Whitney non-parametric parametric test. CCl28 levels in the supernatant of cultured fibroblasts

(proliferating or replication-induced senescecent) (**E**) from normal (n=4) and IPF (n=6) patients. ****p ≤ 0.0001 via one-way ANOVA with Tukey's multiple comparisons test.

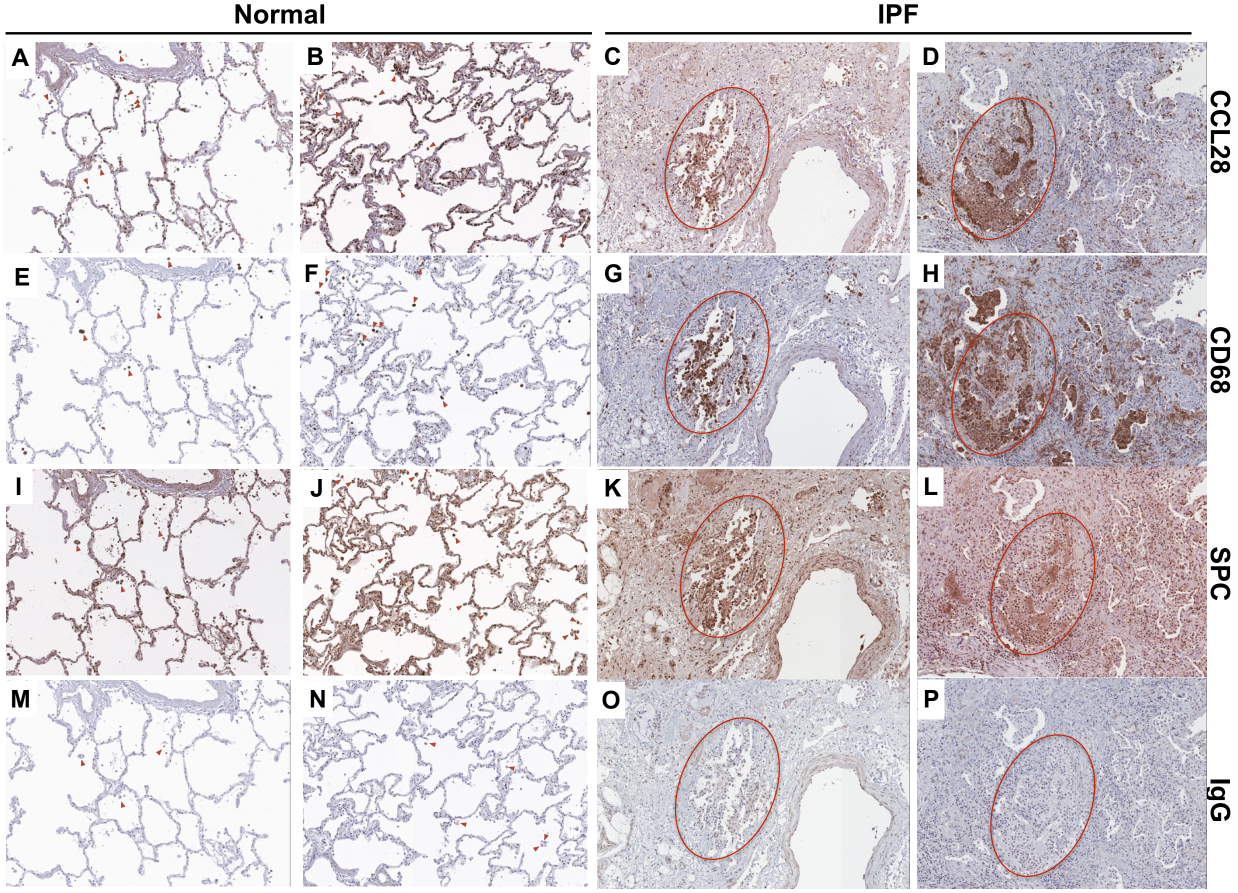

Supplemental Figure 3. CCL28 protein expression in normal and IPF lungs.

Representative images of CCL28 (**A-D**), CD68 (**E-H**), SPC (**I-L**), and isotype IgG (**M-P**) staining in normal (**A-B**, **E-F**, **I-J** and **M-N**) and IPF (**C-D**, **G-H**, **K-L** and **O-P**) lung explants. Shown are images taken at 200x magnification. n=7/group.

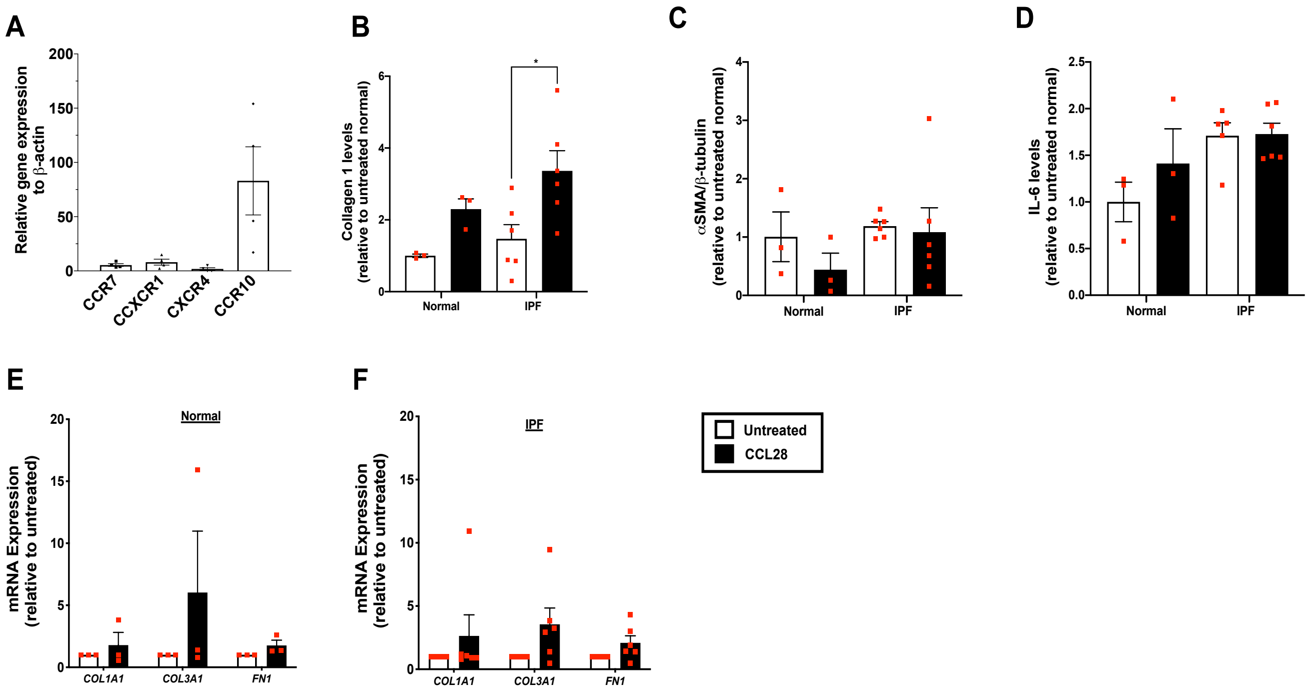

Supplemental Figure 4. **IPF lung fibroblasts express CCR10 and CCL28 induces minor activation**

(**A**) Average expression of *CCR7, CXCR1, CXCR4, and CCR10* transcripts relative to β-actin in IPF lung fibroblasts. Fold change in secreted Collagen 1 (**B**), αSMA/β-tubulin expression (**C**), IL-6 levels (**D**), and fibrosis-related transcripts *COL1A1*, *COL3A1*, and *FN1* in cultured fibroblasts from normal (**E**) or IPF (**F**) patients treated with CCL28 (200 ng/mL) for 72 hours. Data shown are mean ± s.e.m.; n= 3-6 fibroblast lines/group. *p ≤ 0.05 via one-way ANOVA with Tukey's multiple comparisons test.

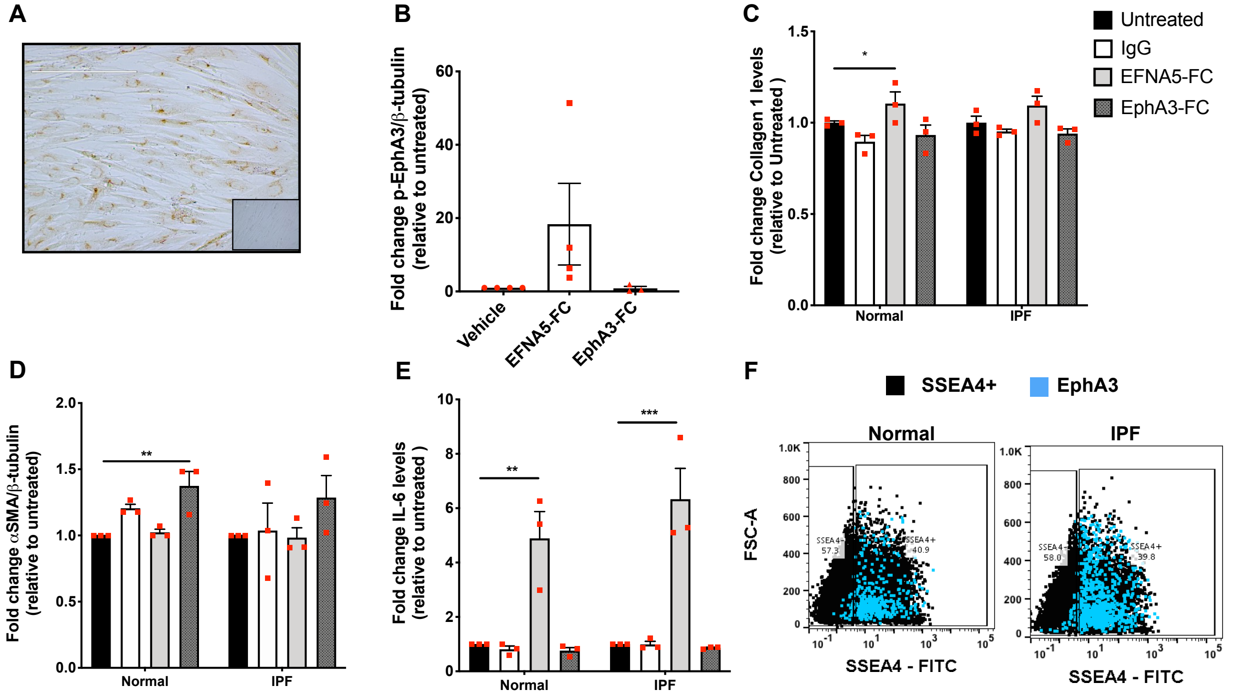

Supplemental Figure 5. Ephrin A ligand, EFNA5, activates EphA3 and promotes Collagen 1 and IL-6 secretion by lung fibroblasts.

(A) Representative image of EphA3 staining (brown) in IPF lung fibroblasts (inlay = isotype control antibody). **(B)** Fold change of p-EphA3/ß-tubulin protein in fibroblast 30 minutes post stimulation with pre-clustered EFNA5-FC (100 nM) with or without Ephrin A ligand neutralizing EphA3-Fc (200 nM). **(C-F)** Lung fibroblasts were treated with IgG control or pre-clustered EFNA5-FC with or without Ephrin A ligand neutralizing EphA3-Fc for 72 hours and shown is the average fold change in (**C**) soluble collagen 1, (**D**) αSMA, and (**E**) IL-6 protein levels in normal and IPF fibroblasts. (**F**) Flow cytometric dot plots of EphA3 staining (Blue) (APC conjugated anti-EphA3) on gated SSEA4^{+} (FITC conjugated streptavidin & biotinylated anti-SSEA4) cells in cultured normal and IPF lung fibroblasts. Data shown are mean ± s.e.m.; n= 3-4 fibroblast lines/group. $*p \leq 0.05$; $**p \leq 0.01$; $***p \leq 0.001$ via one-way ANOVA test with Tukey's multiple comparisons test.

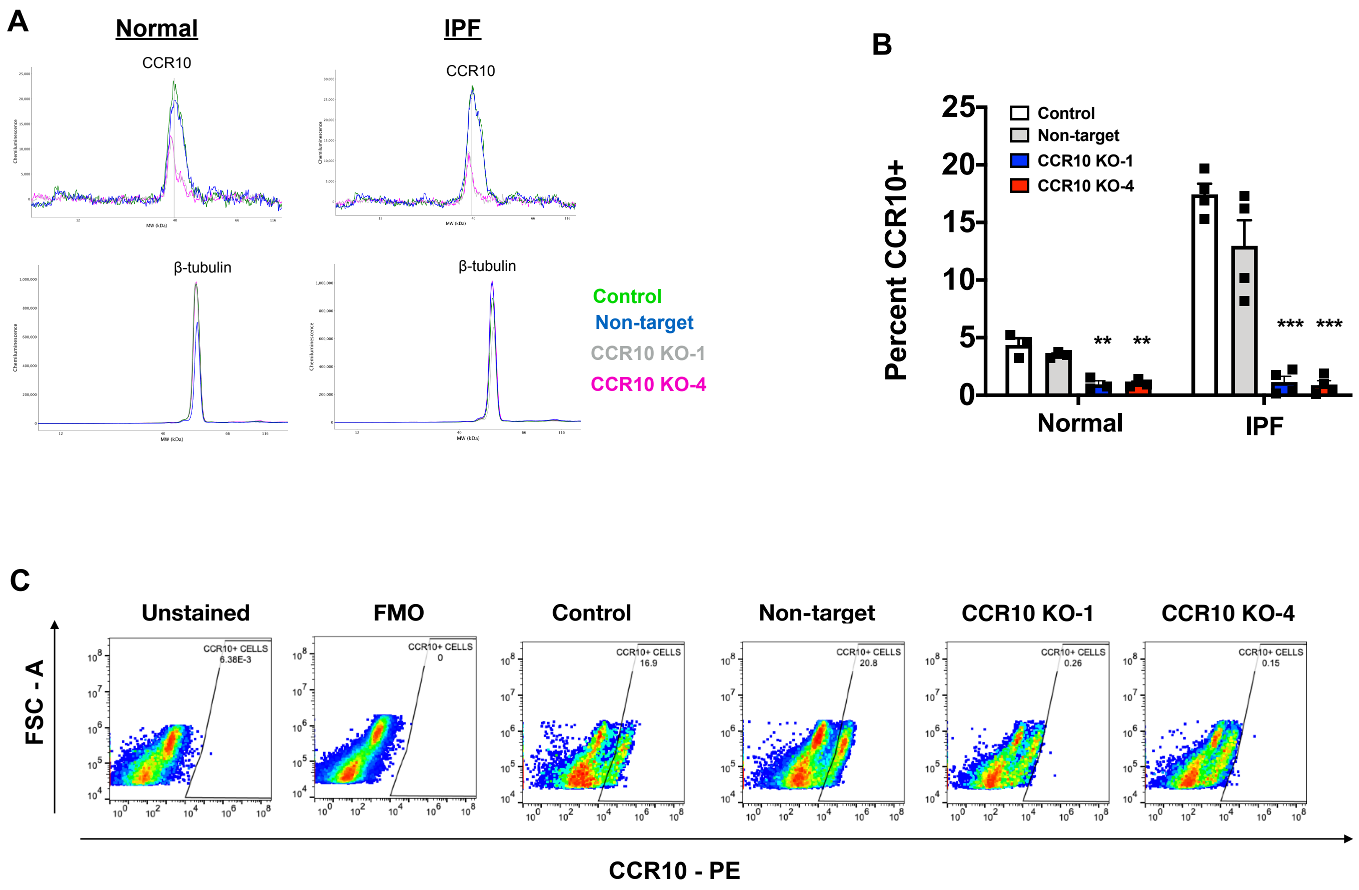

Supplemental Figure 6. Validation of CRISPR-Cas9-mediated targeting of CCR10. **(A)** Representative electropherogram peaks for CCR10 and β-tubulin in control, **(B)** percent CCR10+ cells and **(C)** representative flow cytometric dot plots of CCR10 (PE) expressing cells in control, non-target, and CCR10 KO-1 and 4 fibroblasts, as well as flow cytometric controls. Data shown are mean ± s.e.m.; n= 3-4 fibroblast lines/group. **p ≤ 0.01; ***p ≤ 0.001 via one-way ANOVA test with Tukey's multiple comparisons test.

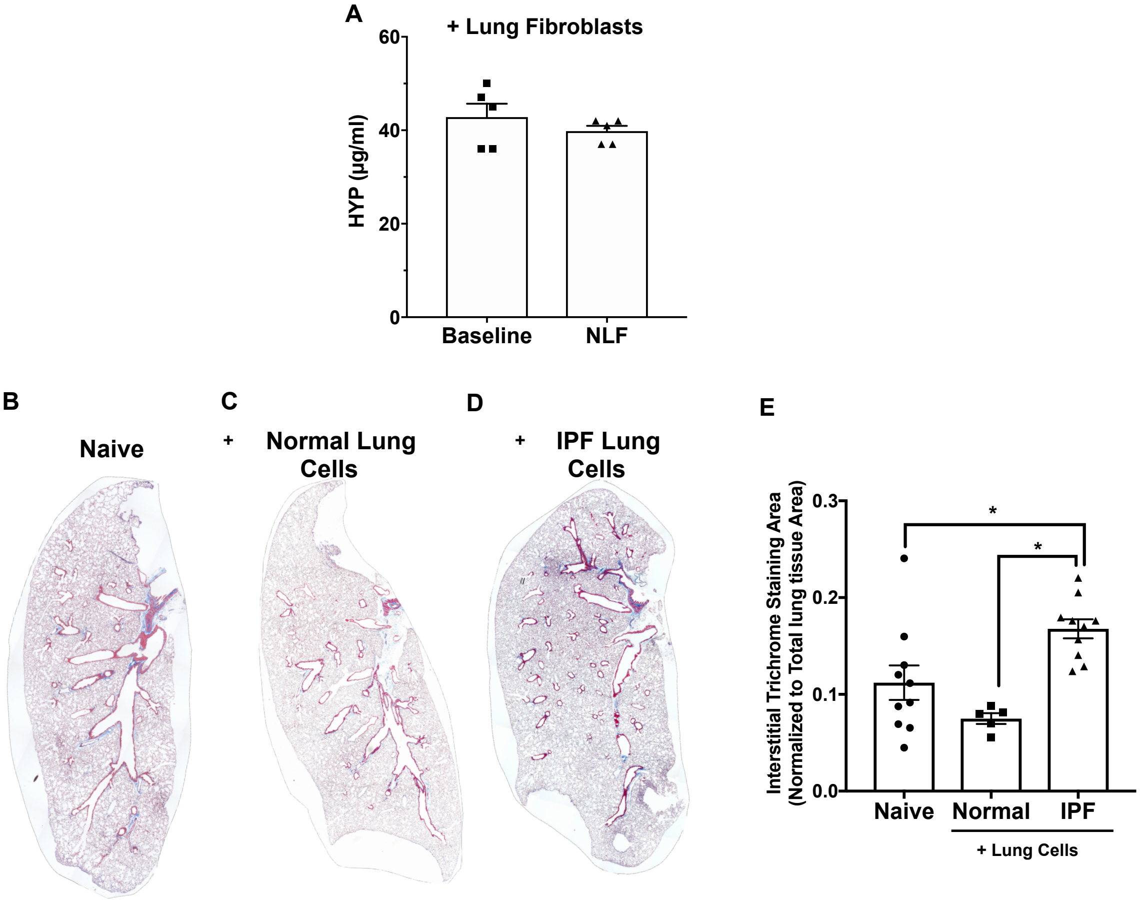

Supplemental Figure 7. Normal lung fibroblasts and explant cells do not promote interstitial fibrosis in the lungs of humanized NSG mice.

(**A**) One million cultured normal lung fibroblasts were I.V. administered into SCID-beige mice. Sixty-three days after cellular administration, mice were sacrificed, and their lungs were biochemically assessed for hydroxyproline concentration. Shown is the mean hydroxyproline concentration in the lungs of naïve and normal lung fibroblast challenged mice. (**B-D**) Shown are whole mount Masson’s trichrome stained non-humanized (**B**), normal lung explant cell-humanized (**C**) or IPF lung explant cell humanized and control IgG treated (**D**) NSG lungs, 63 days after human cell administration. (**E**) Shown are ratios

of the Area sum of interstitial blue trichrome staining normalized to total lung tissue area. n=10 naïve; n=5 normal lung humanized; n=10 IPF lung humanized, IgG control treated NSG lungs. Data shown are mean ± s.e.m.; *$p \leq 0.05$ via via one-way ANOVA test with Tukey's multiple comparisons test.

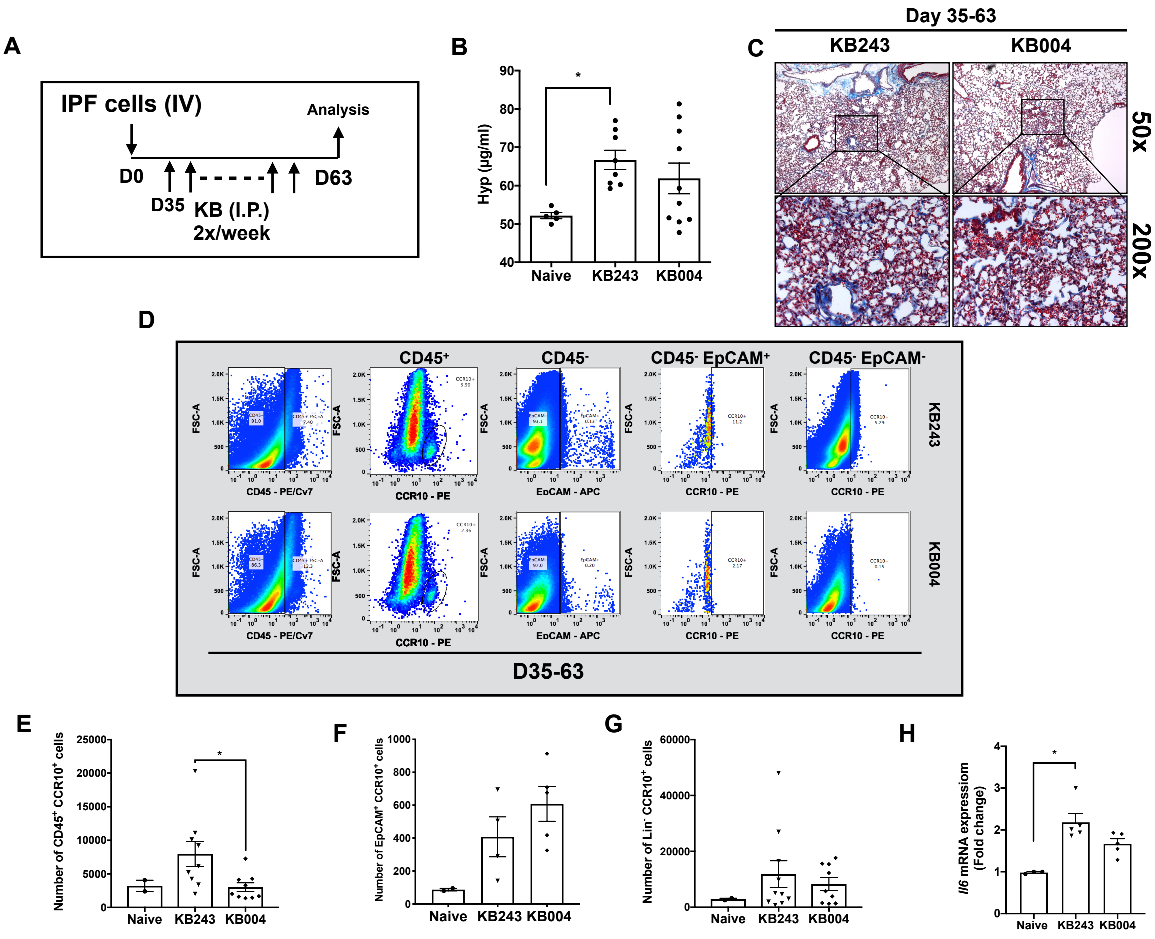

Supplemental Figure 8. Therapeutic administration of KB004 did not reduce established pulmonary fibrosis in humanized NSG mice.

(A) Experimental scheme. **(B)** Hydroxyproline in lungs from humanized NSG mice treated with either KB243 or KB004 mAbs. Data shown are mean ± s.e.m.; n=5-10/group. *p ≤ 0.05 via one-way ANOVA test with Tukey's multiple comparisons test. **(C)** Representative images of Masson's trichrome staining of humanized NSG mouse lung at day 63 after IPF cell injection and therapeutic (D35-63) treatment with either KB243 or KB004. Shown are images taken at 50x (top) and 200x (bottom) magnification. **(D)** From left to right, human $CD45^+$, $CD45^+$ $CCR10^+$, $CD45^-$ $EpCAM^+$, $CD45^-$ $EpCAM^+$ $CCR10^+$, and Lin^-

$CCR10^+$ cells in NSG mouse lungs at day 63 after IPF cell injection and treatment with either KB243 (top) or KB004 (bottom); n=4-5/group. **(E-G)** Depicted is the average number of $CD45^+$ $CCR10^+$ (**E**), $EpCAM^+$ $CCR10^+$ (**F**), and Lin^- $CCR10^+$ (**G**) cells and fold change in the levels of mouse *Il6* mRNA (**H**) in NSG lungs at day 63 after IPF cell injection and therapeutic (D35-63) treatment with either KB243 or KB004. Data shown are mean ± s.e.m.; n=4-5/group. *p ≤ 0.05 via via one-way ANOVA test with Tukey's multiple comparisons test.

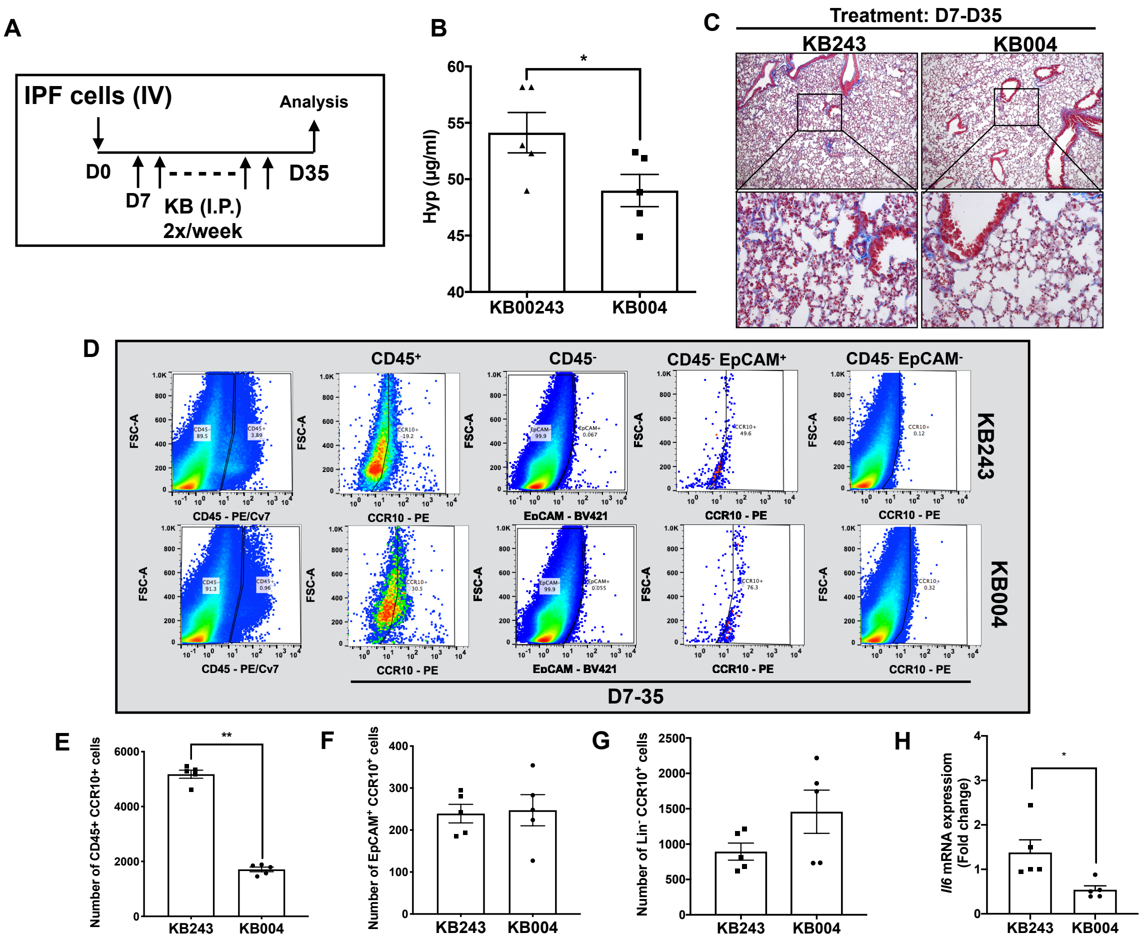

Supplemental Figure 9. Early administration of KB004 significantly ameliorated lung remodeling in humanized NSG mice.

(A) Experimental scheme. **(B)** Hydroxyproline in lungs from humanized NSG mice therapeutically (D7-35) treated with either KB243 or KB004 mAbs. Data shown are mean ± s.e.m.; n=5/group *p ≤ 0.05 via one-tailed Mann Whitney non-parametric test. **(C)** Representative images of Masson's trichrome staining of humanized NSG mouse lung at day 35 after IPF cell injection and therapeutic (D7-35) treatment with either KB243 or KB004. Shown are images taken at 50x (top) and 200x (bottom) magnification. **(D)** From left to right, human $CD45^+$, $CD45^+$ $CCR10^+$, $CD45^-$ $EpCAM^+$, $CD45^-$ $EpCAM^+$ $CCR10^+$, and Lin^- $CCR10^+$ cells in NSG mouse lungs at day 35 after IPF cell injection and

therapeutic (D7-35) treatment with either KB243 or KB004; n=4-5/group. **(E-G)** Human $CD45^+$ $CCR10^+$ (**E**), $EpCAM^+$ CCR10 (**F**), and Lin^- $CCR10^+$ (**G**) cells and fold change in the levels of mouse *Il6* mRNA (**H**) in NSG mice at day 35 after IPF cell injection and therapeutic (D7-35) treatment with either KB243 or KB004. Data shown are mean ± s.e.m.; n=4-5/group. $^{**}p \le 0.01$ via two-tailed Mann Whitney non-parametric test.

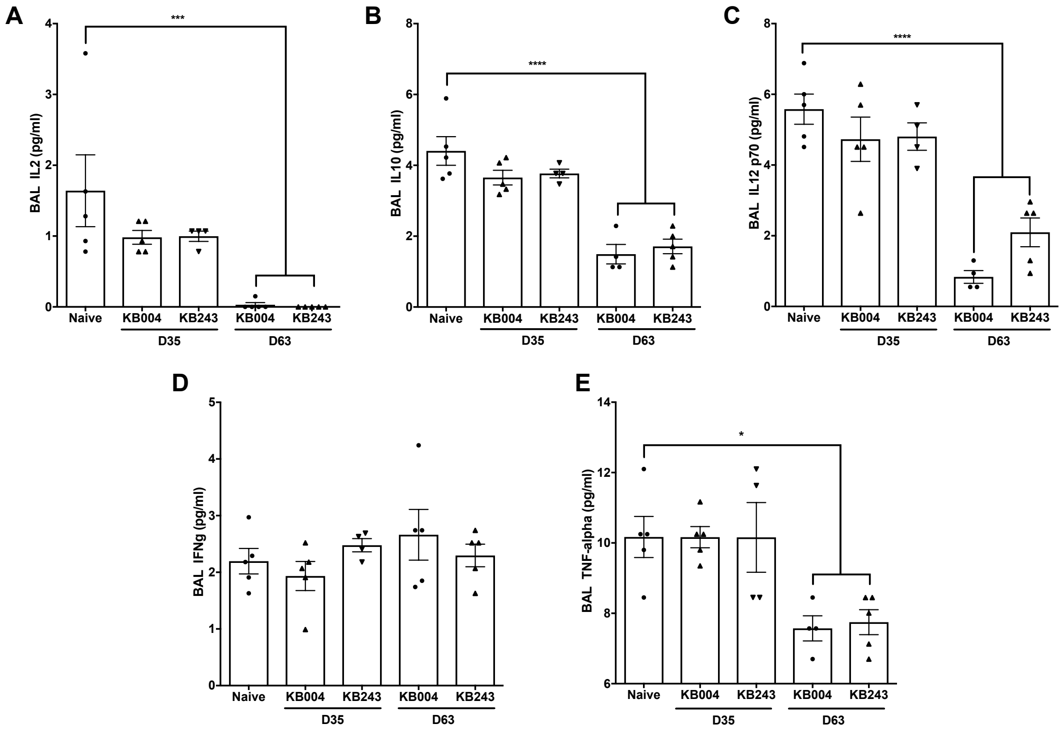

Supplemental Figure 10. IPF lung cells did not promote inflammation in the NSG mouse.

(A-E) Murine IL-2 (**A**), IL-10 (**B**), IL12-p70 (**C**), IFNγ, and TNFα in the BAL from non-humanized and humanized NSG mice treated with KB004 or KB243 from days 0 to 35, or from days 35 to 63 after IPF cell injection. Mouse cytokines were measured using Bioplex and data shown are mean ± s.e.m.; n=5-10/group. *p ≤ 0.05; ***p ≤ 0.001 ****p ≤ 0.0001 via one-way ANOVA with Dunnett correction.

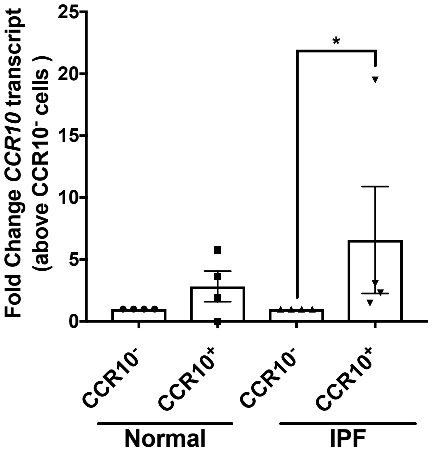

Supplemental Figure 11. Enrichment for CCR10 transcript in magnetically sorted $CCR10^+$ cells.

Flow cytometry antibodies for CCR10 were validated by magnetic antibody-mediated purification of CCR10 expressing (i.e. $CCR10^+$) cells from normal and IPF lung explants followed by RNA extraction and qPCR analysis for the chemokine receptor in the sorted cells. Depicted is the average expression of CCR10 transcript in the sorted cells compared with non-sorted cells. Data are mean ± sem; n=4. *$p \leq 0.05$ via two-tailed Mann Whitney non-parametric test.

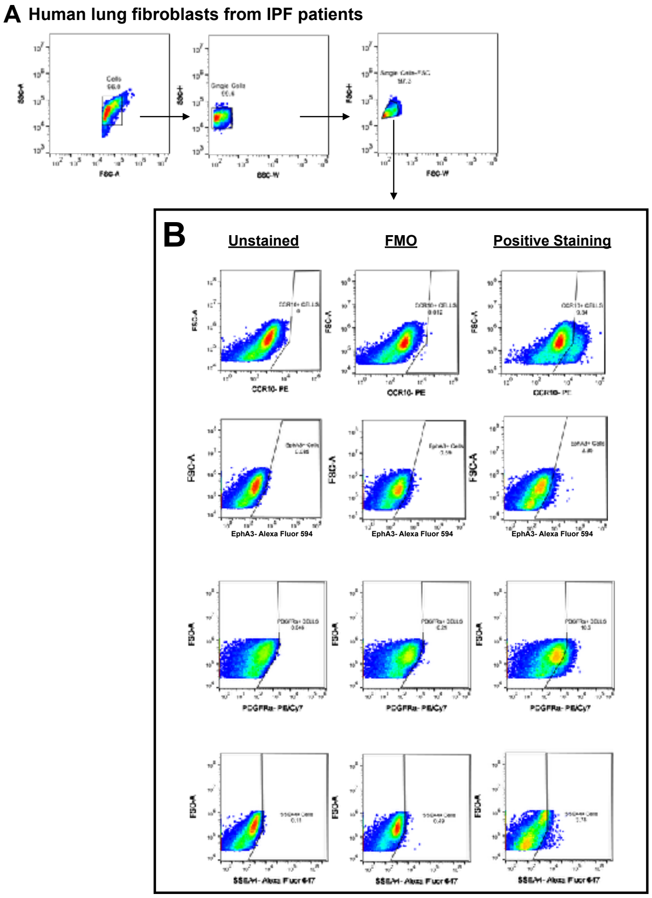

Supplemental Figure 12. Flow cytometric gating strategy of human lung fibroblasts.

(A) Gating of human lung fibroblasts from IPF patients shown as a representation of the gating applied for flow cytometric analysis of normal and IPF human lung fibroblast. **(B)** Depicted are representative flow cytometric dot plots for human CCR10, EpHA3, SSEA4, and PDGFRα gates that were determined based upon respective flow cytometric unstained and staining controls (Fluorescence minus one (FMO) and positive staining).

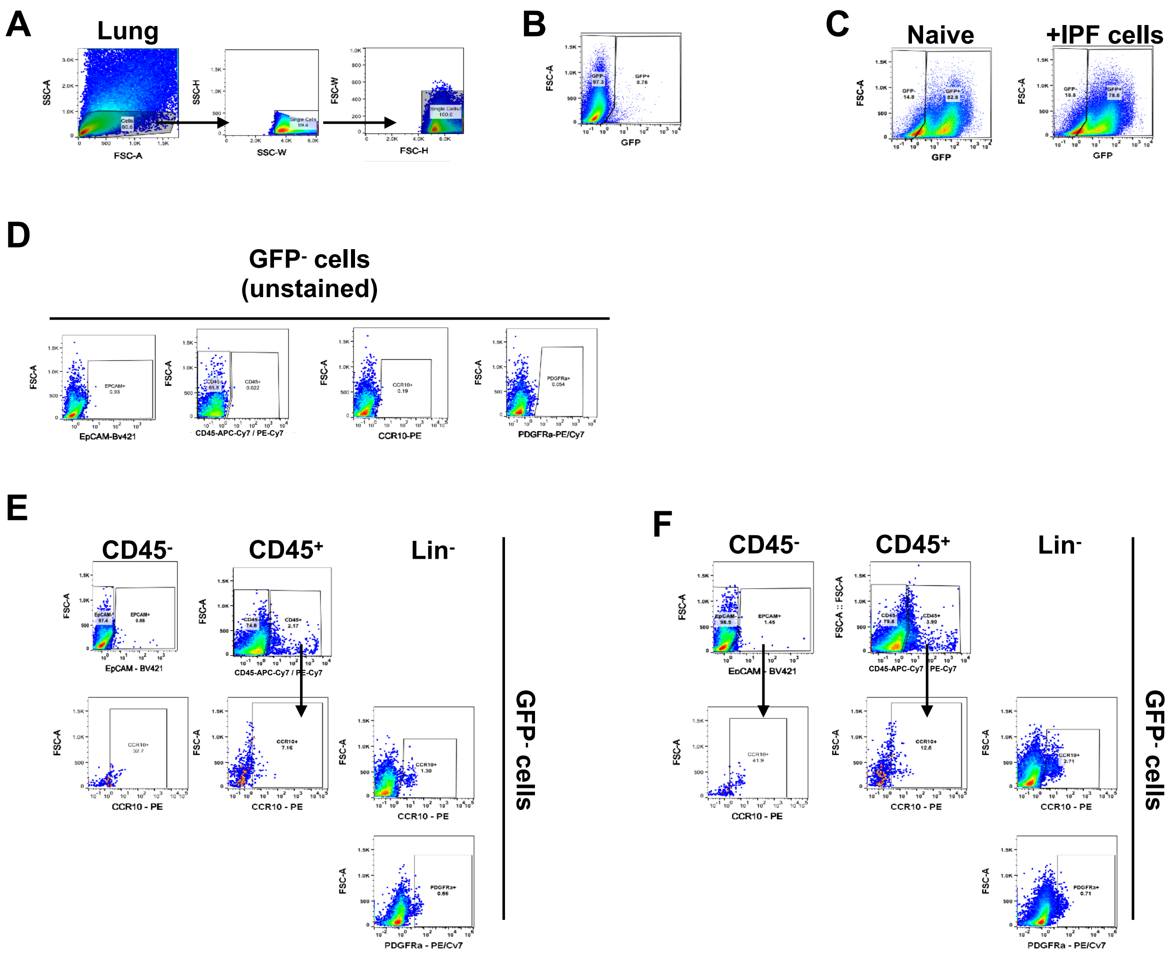

Supplemental Figure 13. Flow cytometric gating strategy of human cells in the lungs of GFP-NSG mice.

Human GFP$^-$ cells were detected in the lungs of GFP-NSG mice at day 35 after IPF cell injection using flow cytometry. **(A)** Gating of cells of interest. **(B)** GFP$^-$ mouse cells from wildtype mice were used to determine both the GFP$^+$ and GFP$^-$ gates. **(C-D)** Human CD45, EpCAM, CCR10, and PDGFRα gates were determined based upon any background staining observed with the anti-human antibodies in non-humanized mice and respective flow cytometry controls. Depicted are representative flow cytometric dot plots for various human cell types present in GFP-NSG the lungs of naïve mice **(E)** or mice that received IPF cells 35 days previously **(F)**.

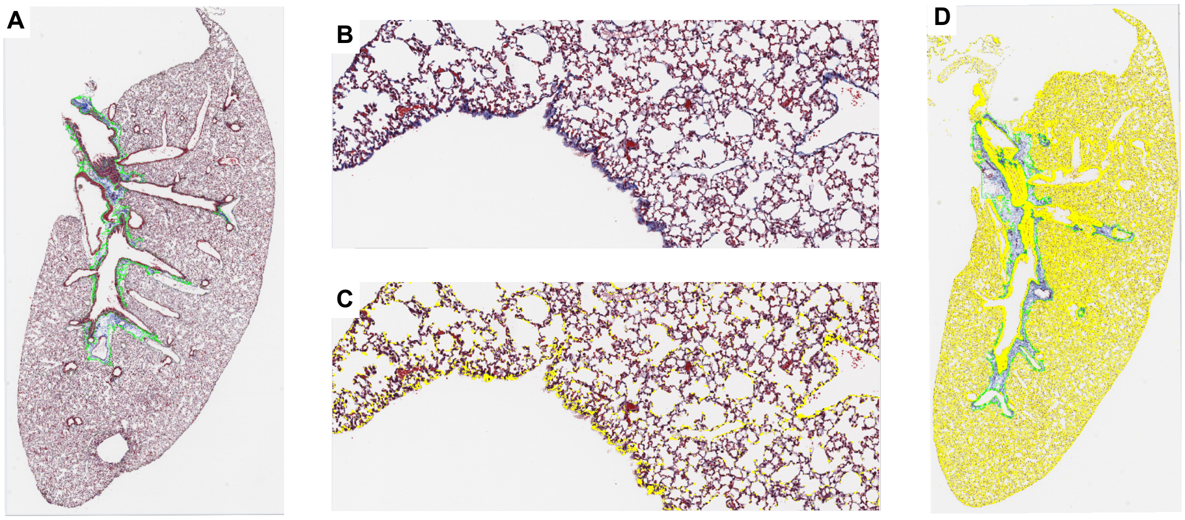

Supplemental Figure 14. Quantification of interstitial Masson's Trichrome-blue staining.

Lung tissues were Masson's trichrome stained and tile images of the stained NSG lungs were acquired using a Zeiss AX10 microscope, stitched and exported using Zen 2012 (blue edition) v 1.1.2.0 software. Images were then imported into Image pro premier v9.3.3. (**A-C**) Using image pro, regions of interest (ROI) around large airways and vessels were created (based on the size and blue color of these regions; and the area sum of interstitial trichrome blue staining (**B**) was quantified outside these ROIs (yellow overlay, **C**). This was then normalized to the total area of lung tissue outside the ROIs (yellow overlay, **C**).

Supplemental Table 1: IPF patient demographics.

Patient ID	Age	Sex	Diagnosis	Progression	Therapy at time of explant	Analysis
IPF09-14	71	Male	IPF	Slow	Participated in PRM-151 trial	NSG mice, Flow Cytometry, in vitro analysis
IPF10-14	70	Male	IPF	Unknown	Unknown	Flow Cytometry
IPF11-14	73	Male	IPF	Unknown	Unknown	Flow Cytometry, in vitro analysis
IPF12-14	52	Male	IPF	Unknown	Unknown	Flow Cytometry
IPF14-14	64	Male	IPF	Rapid	N-acetylcysteine	NSG mice, Flow Cytometry, in vitro analysis
IPF01-15	72	Male	IPF	Unknown	Unknown	Flow Cytometry
IPF02-15	64	Male	IPF	Unknown	cefepime, azithromycin, slow steroid taper with Bactrim prophylaxis and Pirfenidone taper	NSG mice, Flow Cytometry, in vitro analysis
IPF03-15	68	Female	IPF	Unknown	Unknown	NSG mice, Flow cytometry
IPF04-15	67	Male	IPF	Unknown	Pirfenidone	Flow Cytometry, in vitro analysis
IPF06-15	72	Female	IPF	Unknown	Unknown	Flow Cytometry
IPF01-16	58	Female	IPF	Unknown	Unknown	Flow Cytometry
IPF02-16	58	Male	IPF	Unknown	Nintedanib	Flow Cytometry, in vitro analysis
ILD03-16	58	Male	IPF	Unknown	Unknown	Flow Cytometry, in vitro analysis
IPF04-18	65	Female	IPF	Unknown	Unknown	Flow Cytometry, in vitro analysis
IPF05-18	65	Male	IPF	Unknown	Unknown	Flow Cytometry, in vitro analysis
IPF08-18	49	Female	IPF	Unknown	Unknown	Flow Cytometry, in vitro analysis
IPF09-18	64	Male	IPF	Unknown	Unknown	Flow Cytometry, in vitro analysis
IPF10-18	66	Male	IPF	Unknown	Unknown	In vitro analysis
IPF11-18	61	Female	IPF	Unknown	Unknown	In vitro analysis
IPF15-18	65	Female	IPF	Unknown	Unknown	In vitro analysis

IPF01-19	65	Female	IPF	Unknown	Unknown	Flow Cytometry, in vitro analysis
IPF04-19	64	Male	IPF	Unknown	Unknown	In vitro analysis
IPF07-19	69	Male	IPF	Unknown	Unknown	In vitro analysis

Supplemental Table 2. Transcript expression of fibrosis-related tyrosine kinases that are not targeted by nintedanib in IPF lung explants

**Transcript**	**Fold Change**	**p value**
EPHA3	10.622	0.000019
DDR1	3.312	0.00000227
NTRK2	3.242	0.0278
PTK2	2.81	0.00000954
ERBB3	2.376	0.000935
ERBB2	2.252	0.0000766
STYK1	2.216	0.0133
EPHB3	2.036	0.0000573
TNK2	1.965	0.00278
CDK1	1.901	0.0105
CSF1R	1.876	0.0124
PTK7	1.844	0.00175
RET	1.79	0.000109
ROR2	1.759	0.00456
ZAP70	1.652	0.0568
PTK6	1.633	0.00565
MST1R	1.605	0.00379
EPHB2	1.57	0.0113
ERB4	1.473	0.058
TYK2	1.427	0.00752

EPHA2	1.388	0.181
PTK2B	1.376	0.119
DDR2	1.354	0.31
TIE1	1.343	0.297
LMTK3	1.343	0.138
MATK	1.337	0.0171
EPHB6	1.336	0.201
KIT	1.278	0.425
EPHA1	1.261	0.295
MET	1.241	0.49
ABL1	1.221	0.295
AXL	1.216	0.278
ITK	1.206	0.565
BTK	1.204	0.436
EPB1	1.182	0.174
RYK	1.14	0.339
EPHB4	1.137	0.238
CDK4	1.137	0.327
TNK1	1.105	0.469
TXK	1.098	0.635
NTRK1	1.061	0.669
INSRR	1.048	0.62
CSK	1.004	0.975
HCK	-1.041	0.925
FRK	-1.05	0.878

SRMS	-1.064	0.484
FES	-1.071	0.81
IGF2R	-1.106	0.72
TYRO3	-1.132	0.35
TEC	-1.133	0.327
FGR	-1.135	0.681
EPHA8	-1.144	0.315
ABL2	-1.201	0.447
EPHA10	-1.208	0.149
ALK	-1.258	0.191
EPHA6	-1.267	0.141
MUSK	-1.267	0.104
AATK	-1.292	0.0824
EPHA7	-1.34	0.0603
LTK	-1.345	0.218
EPHA4	-1.396	0.171
EPHA5	-1.421	0.0257
INSR	-1.425	0.0799
BLK	-1.452	0.0135
JAK2	-1.545	0.0329
ROS1	-1.548	0.171
FER	-1.549	0.0159
SYK	-1.559	0.0333
JAK3	-1.584	0.148
MERTK	-1.618	0.0908

YES1	-1.733	0.00758
IGF1R	-1.747	0.0371
CDK2	-1.954	0.00915
LMTK2	-2.04	0.0006
EGFR	-2.041	0.00611
NTRK3	-2.056	0.0197
FLT3	-2.146	0.000138
FYN	-2.25	0.000848
ROR1	-2.44	0.00638
TEK	-2.803	0.0041
JAK1	-2.958	0.000982

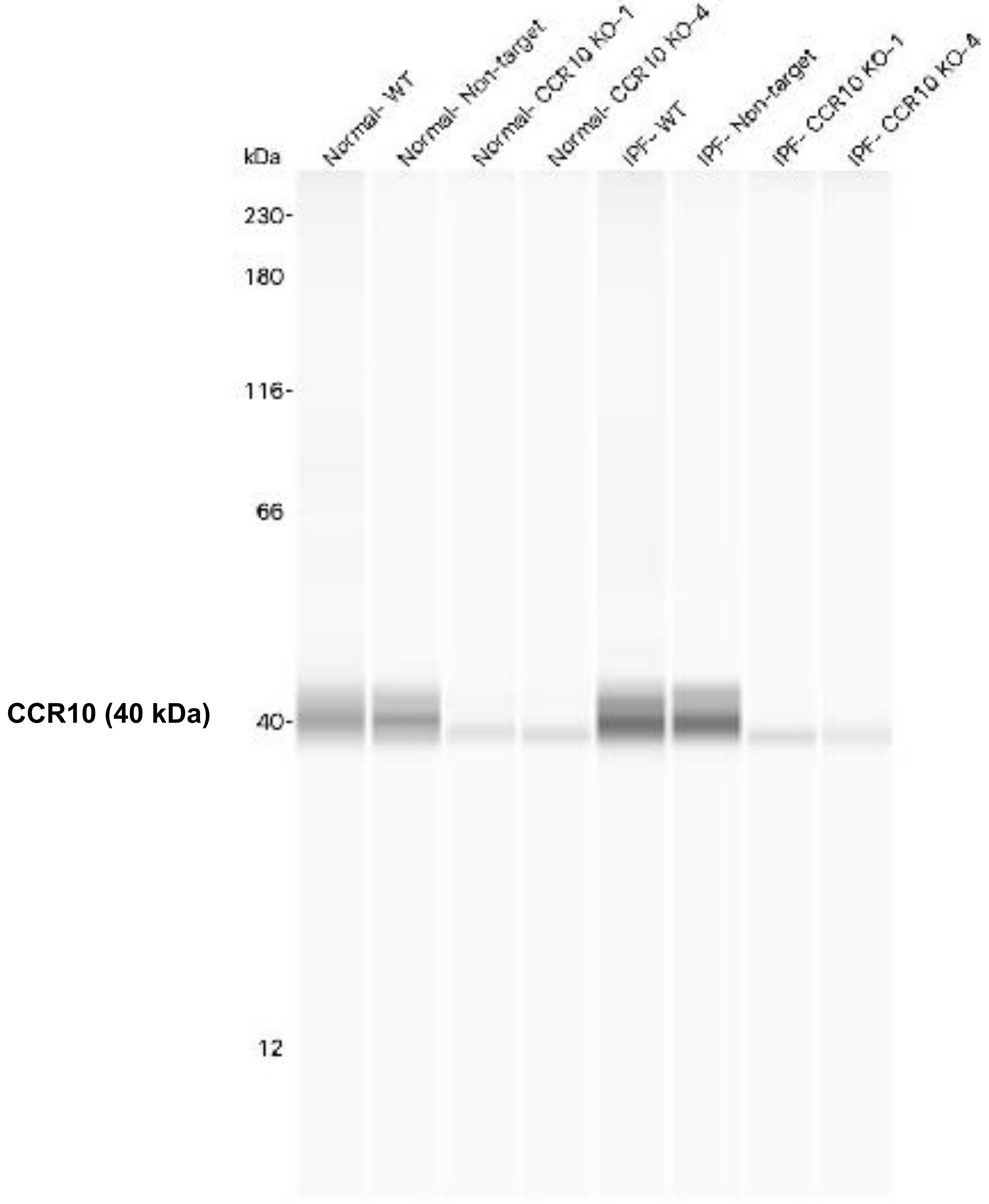

CCR10 (40 kDa)
kDa
230-
180
116-
66
40-
12
Normal- WT
Normal- Non-target
Normal- CCR10 KO-1
Normal- CCR10 KO-4
IPF- WT
IPF- Non-target
IPF- CCR10 KO-1
IPF- CCR10 KO-4

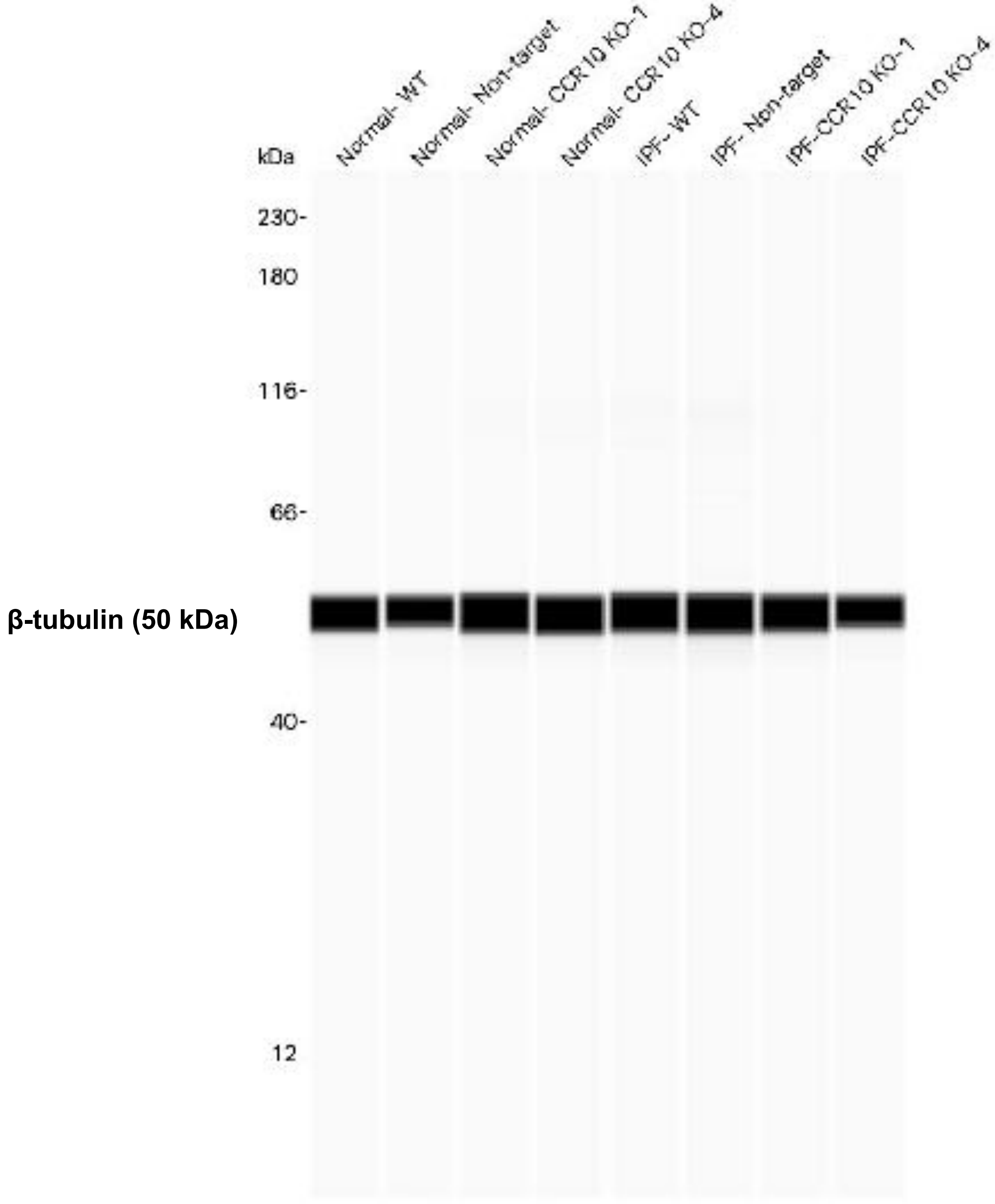

β-tubulin (50 kDa)
kDa
230-
180
116-
66-
40-
12
Normal- WT
Normal- Non-target
Normal- CCR10 KO-1
Normal- CCR10 KO-4
IPF- WT
IPF- Non-target
IPF-CCR10 KO-1
IPF-CCR10 KO-4
